# Supplementary material for: Are you with me? Co-occurrence tests from community ecology can identify positive and negative epistasis between inversions in Mimulus guttatus
Source: PLoS One. 2025 Apr 28;20(4):e0321253. doi: 10.1371/journal.pone.0321253 (PMC12036897; doi:10.1371/journal.pone.0321253)
Supplement: S1 Word Doc — (DOCX) [file pone.0321253.s005.docx]

**Are you with me? Co-occurrence tests from community ecology can identify positive and negative epistasis between inversions in *Mimulus guttatus***

Luis J. Madrigal-Roca*^1^ & John K. Kelly^1^

**^1^** Ecology and Evolutionary Biology’s Department of the University of Kansas, Haworth Hall, Room 5007, 1200 Sunnyside Ave, Lawrence, Kansas, United States of America.

Corresponding author: Luis J. Madrigal-Roca

*Email: [madrigalrocalj@yahoo.com](mailto:madrigalrocalj@yahoo.com)

**EXTENDED METHODS**

Affinity score

The affinity score proposed by Mainali *et al*. (2022) was one of the ecological measures adopted in this study. It is a measure formally defined as the following log odds ratio

$$\alpha=log({\frac{p_{1}}{1-p_{1}}}/{\frac{p_{2}}{1-p_{2}}})$$

where the parameter $\alpha$ describes the affinity of the entities under consideration (inversion *loci* in this study) or the tendency to co-occur (coupling state). Taking as a reference one of such entities in a pair, that entities will be present with $p_{1}$ if the other inversion is present and with $p_{2}$ if the other inversion is absent. The conditional co-occurrence or coupling distribution has the general shape described be the hypergeometric distribution when $\alpha=0$, but it can be formalized as follows

$$P\left( X=k \right)={\binom{m_{A}}{k}\binom{N-m_{A}}{m_{B}-k}e^{\alpha k}}/{\sum_{j=0}^{m_{B}} \binom{m_{A}}{j}}\binom{N-m_{A}}{m_{B}-j}e^{\alpha j}$$

In the previous relation, $N$ refers to the number of sites (in ecology) or individuals (in the genetic context, these are the units where *loci* are genotyped), of which $m_{A}$ contains the entity 1 (inversion 1) and $m_{B}$ the entity 2 (inversion 2). $k$ is the count of cases where both entities appear together. If the two entities under consideration are in coupling more often than described by the expectation of the hypergeometric distribution, we expect the log odds ratio $\alpha$ to be positive. Similarly, repulsion between the entities under consideration is associated with a negative value of $\alpha$.

Centered Jaccard-Tanimoto index

The Centered Jaccard-Tanimoto index proposed by Chung *et al*. (2019) was the other measure of co-occurrence used in the context of this research. This measure is based on the original Jaccard-Tanimoto index, or intersection to union ratio of the two entities under analysis.

$$J(y_{i},y_{j})={y_{i}\cap y_{j}}/{y_{i}\cup y_{j}}$$

Where $y_{i}$ and $y_{j}$ represent the presence / absence vector of entities *i* and *j*, respectively (inversion in our case). Under the null model, those entities are assumed to be independent, and their occurrences are modelled following a Bernoulli distribution. Hence, the expected value for the Jaccard-Tanimoto index is

$$\mathbb{E}\left( J\left( y_{i},y_{j} \right) \right)= {p_{i}p_{j}}/{p_{i}+p_{j}-p_{i}p_{j}}$$

Where $p_{i}$ is the probability of occurrence of the first entity and $p_{j}$ is the probability of occurrence of the second entity. So, given a pair of entities, we can calculate the Centered Jaccard-Tanimoto index by subtracting the null expectation from the observed index as follows

$$J^{C}\left( y_{i},y_{j} \right)=J\left( y_{i},y_{j} \right)-\mathbb{E}\left( J\left( y_{i},y_{j} \right) \right)$$

This index naturally distinguishes between positive ($J^{C}>0$) or negative associations ($J^{C}<0$), being the null hypothesis that such measure is equal to 0 (independence). All the estimation for the significance of the centered index is described in the original paper, and in general, it can follow four methods: exact, asymptotic, measure concentration algorithm (MCA), and bootstrap.

Comparison between LD and co-occurrence metrics in an allele-based analysis

To evaluate the performance and properties of different association metrics, we conducted a simulation study comparing traditional linkage disequilibrium (LD) measures (D and D') with co-occurrence metrics (centered Jaccard-Tanimoto index and Affinity). We simulated genotype data for pairs of genetic *loci* across different allele frequency combinations and a fixed association strength.

Genotype data was simulated using a copula-based approach to generate correlated binary outcomes, which were then converted to genotype counts (0, 1, or 2 alternative alleles) following Hardy-Weinberg proportions. In order to do so, we used the copula R package [3–6]. We used allele frequencies ranging from 0.1 to 0.9 (in steps of 0.1) for both *loci*, creating a grid of frequency combinations. For each frequency combination, we generated 1,000 replicates of 400 samples each, with a fixed association strength of 0.5.

We assessed the frequency dependence and stability of each metric through several approaches:

1. Relative variation across frequencies, calculated as the ratio of standard deviation to mean of median values across frequency combinations.

2. Complexity of frequency relationships estimated using the effective degrees of freedom (EDF) from generalized additive models.

3. Direct correlations between metrics and allele frequencies.

4. Heatmap plot showing the values for the metric per combination of the inversion frequencies for the same association strength. For this plot, a desire property will be homogeneous distribution and reduced vcariability of values for the statistics, considering that the association strength is fixed.

All analyses were performed in R (version 4.4.2) [7], using the mgcv package [8–12] for generalized additive modeling and custom functions for metric calculations.

**REFERENCES**

1. Mainali KP, Slud E, Singer MC, Fagan WF. A better index for analysis of co-occurrence and similarity. Sci Adv. 2022 Jan 28;8(4):eabj9204.

2. Chung NC, Miasojedow B, Startek M, Gambin A. Jaccard/Tanimoto similarity test and estimation methods for biological presence-absence data. BMC Bioinformatics. 2019 Dec;20(S15):644.

3. Marius Hofert, Martin Mächler. Nested Archimedean Copulas Meet R: The nacopula Package. Journal of Statistical Software. 2011;39(9):1–20.

4. Ivan Kojadinovic, Jun Yan. Modeling Multivariate Distributions with Continuous Margins Using the copula R Package. Journal of Statistical Software. 2010;34(9):1–20.

5. Jun Yan. Enjoy the Joy of Copulas: With a Package copula. Journal of Statistical Software. 2007;21(4):1–21.

6. Hofert M, Kojadinovic I, Maechler M, Yan J. copula: Multivariate Dependence with Copulas [Internet]. 2024. Available from: https://CRAN.R-project.org/package=copula

7. R Core Team. R: A Language and Environment for Statistical Computing [Internet]. Vienna, Austria: R Foundation for Statistical Computing; 2024. Available from: https://www.R-project.org/

8. Wood SN. Generalized Additive Models: An Introduction with R. 2nd ed. Chapman and Hall/CRC; 2017.

9. Wood SN. Thin-plate regression splines. Journal of the Royal Statistical Society (B). 2003;65(1):95–114.

10. Wood SN. Stable and efficient multiple smoothing parameter estimation for generalized additive models. Journal of the American Statistical Association. 2004;99(467):673–86.

11. Wood SN, N., Pya, S"afken B. Smoothing parameter and model selection for general smooth models (with discussion). Journal of the American Statistical Association. 2016;111:1548–75.

12. Wood SN. Fast stable restricted maximum likelihood and marginal likelihood estimation of semiparametric generalized linear models. Journal of the Royal Statistical Society (B). 2011;73(1):3–36.
