# Supplementary material for: Are you with me? Co-occurrence tests from community ecology can identify positive and negative epistasis between inversions in Mimulus guttatus
Source: PLoS One. 2025 Apr 28;20(4):e0321253. doi: 10.1371/journal.pone.0321253 (PMC12036897; doi:10.1371/journal.pone.0321253)
Supplement: S2 Word Doc — (DOCX) [file pone.0321253.s006.docx]

**Are you with me? Co-occurrence tests from community ecology can identify genetic repulsions and attractions in *Mimulus guttatus***

Luis J. Madrigal-Roca & John K. Kelly

**Supporting information**

**S1 Table: Chromosome and crosses where the inversions detected for a natural population of *Mimulus guttatus*, in Iron Mountain, Oregon, are segregating.**

| **Inv** | **Chr** | **Line** |
| --- | --- | --- |
| 1 | Chr_01 | 444 |
| 2 | Chr_01 | 909 |
| 3 | Chr_01 | 1034 |
| 4 | Chr_01 | 155;444;502;909 |
| 5 | Chr_01 | 62 |
| 6a | Chr_01 | 664 |
| 6b | Chr_01 | 444 |
| 7 | Chr_02 | 62;155;444;664;1034;1192 |
| 8 | Chr_02 | 62;541 |
| 9 | Chr_02 | 155;502;541;664;909;1034 |
| 10a | Chr_02 | 1034 |
| 10b | Chr_02 | 62;155;444;541;664 |
| 11 | Chr_02 | 1034 |
| 12 | Chr_03 | 541 |
| 13 | Chr_03 | 444 |
| 14 | Chr_03 | 62 |
| 15 | Chr_03 | 155 |
| 16 | Chr_03 | 155;444 |
| 17 | Chr_04 | 1034;1192 |
| 18 | Chr_04 | 62 |
| 19 | Chr_04 | 909;1192 |
| 20 | Chr_04 | 664 |
| 21 | Chr_05 | 502;664;909 |
| 22 | Chr_05 | 444;502;664 |
| 23 | Chr_05 | 62;1192 |
| 24 | Chr_06 | 664 |
| 25 | Chr_06 | 155;444;909;1034;1192 |
| 26 | Chr_06 | 444 |
| 27 | Chr_07 | 502;541;1192 |
| 28 | Chr_08 | 541;909;1192 |
| 29 | Chr_08 | 62;155;444;502;541;664;909;1034;1192 |
| 30 | Chr_08 | 664 |
| 31 | Chr_08 | 502;909 |
| 32 | Chr_08 | 62;155;444;502;541;664;909;1034;1192 |
| 33 | Chr_08 | 155;541 |
| 34 | Chr_08 | 444 |
| 35 | Chr_08 | 62;155;444;502;541;664;909;1034 |
| 36 | Chr_09 | 62 |
| 37 | Chr_09 | 62;909 |
| 38 | Chr_09 | 1034 |
| 39 | Chr_10 | 62;664;1034 |
| 40 | Chr_10 | 62;155;444;502;541;664;909;1034;1192 |
| 41 | Chr_10 | 1192 |
| 42 | Chr_10 | 1034 |
| 43 | Chr_10 | 444;502;541;664;1034;1192 |
| 44 | Chr_10 | 909 |
| 45 | Chr_10 | 444;502;664;909 |
| 46 | Chr_10 | 909 |
| 47 | Chr_11 | 502;541;909 |
| 48 | Chr_11 | 62;502;541;664;909 |
| 49 | Chr_12 | 62;155;444;502;541;909;1034 |
| 50 | Chr_12 | 664 |
| 51 | Chr_12 | 155;541;664 |
| 52 | Chr_12 | 502 |
| 53 | Chr_13 | 62;155;444;541;909;1034;1192 |
| 54 | Chr_13 | 502;664;909;1192 |
| 55 | Chr_13 | 502;541;1034 |
| 56 | Chr_13 | 444;664 |
| 57 | Chr_14 | 62 |
| 58 | Chr_14 | 155;444;541;1034 |
| 59 | Chr_14 | 62 |
| 60 | Chr_14 | 1034 |
| 61 | Chr_14 | 502;1192 |
| 62 | Chr_14 | 62;155;502;664;909;1034;1192 |

**S2 Table: Significant contrasts according to the raw *p* value that were detected by at least one of the three 2x2 table-based approaches employed to assess the occurrence patterns of pairs of inversions in lines of *Mimulus guttatus* from Iron Mountain, Oregon, USA.** The omnibus $\chi^{2}$ and *p*-values associated to the 3x3 table are also reported here. $\boldsymbol{\chi}^{\boldsymbol{2}}$**_global**: omnibus $\chi^{2}$ value (3x3 table-derived contingency analysis). ***p*_**$\boldsymbol{\chi}^{\boldsymbol{2}}$**_g**: uncorrected *p*-value associated with the omnibus $\chi^{2}$ test. ***p*_**$\boldsymbol{\chi}^{\boldsymbol{2}}$**_ph**: uncorrected *p*-value associated with the $\chi^{2}$ post-hoc test. $\boldsymbol{\chi}^{\boldsymbol{2}}$**_RC**: Relative contribution of the contrast to the omnibus $\chi^{2}$value (percent). $\boldsymbol{\chi}^{\boldsymbol{2}}$**_SR**: Standardized residual of the contrast in relation to the omnibus $\chi^{2}$value. **α**: Affinity score. **α _*p***: uncorrected *p*-value associated to the affinity score. **cJ/T**: Centered Jaccard-Tanimoto score. **cJ/T_*p***: uncorrected *p*-value associated to the Jaccard-Tanimoto score.

| **Cross** | **INV_1** | **INV_2** | **Chr_1** | **Chr_2** | $\chi^{2}$**_global** | ***p*_**$\chi^{2}$**_g** | ***p*_**$\chi^{2}$**_ph** | $\chi^{2}$**_RC** | $\chi^{2}$**_SR** | **α** | **α _*p*** | **cJ/T** | **cJ/T_*p*** |
| --- | --- | --- | --- | --- | --- | --- | --- | --- | --- | --- | --- | --- | --- |
| L_1034 | Inv_10a_1 | Inv_29_2 | Chr_02 | Chr_08 | 10.687 | 0.029 | 0.029 | 16.939 | 2.180 | 0.900 | 0.045 | 0.073 | 0.023 |
| L_1034 | Inv_10a_1 | Inv_49_1 | Chr_02 | Chr_12 | 6.200 | 0.189 | 0.023 | 17.527 | -2.268 | -0.750 | 0.033 | -0.075 | 0.031 |
| L_1034 | Inv_11_1 | Inv_29_1 | Chr_02 | Chr_08 | 8.065 | 0.088 | 0.046 | 10.145 | -1.994 | -0.658 | 0.051 | -0.066 | 0.061 |
| L_1034 | Inv_11_1 | Inv_43_1 | Chr_02 | Chr_10 | 5.759 | 0.221 | 0.020 | 19.273 | -2.322 | -0.770 | 0.023 | -0.077 | 0.026 |
| L_1034 | Inv_11_2 | Inv_38_2 | Chr_02 | Chr_09 | 5.033 | 0.297 | 0.049 | 45.261 | 1.968 | 0.837 | 0.062 | 0.082 | 0.037 |
| L_1034 | Inv_17_1 | Inv_32_1 | Chr_04 | Chr_08 | 7.093 | 0.132 | 0.020 | 16.177 | -2.326 | -0.774 | 0.022 | -0.076 | 0.030 |
| L_1034 | Inv_17_1 | Inv_62_1 | Chr_04 | Chr_14 | 5.284 | 0.264 | 0.049 | 13.154 | 1.968 | 0.654 | 0.066 | 0.070 | 0.044 |
| L_1034 | Inv_17_2 | Inv_55_1 | Chr_04 | Chr_13 | 9.750 | 0.042 | 0.034 | 13.419 | -2.116 | -0.838 | 0.041 | -0.054 | 0.044 |
| L_1034 | Inv_29_2 | Inv_55_1 | Chr_08 | Chr_13 | 4.478 | 0.349 | 0.045 | 26.278 | 2.007 | 0.911 | 0.063 | 0.057 | 0.034 |
| L_1034 | Inv_29_2 | Inv_9_1 | Chr_08 | Chr_02 | 10.687 | 0.029 | 0.029 | 16.939 | 2.180 | 0.900 | 0.045 | 0.073 | 0.022 |
| L_1034 | Inv_32_1 | Inv_38_2 | Chr_08 | Chr_09 | 6.924 | 0.146 | 0.022 | 28.272 | -2.291 | -0.853 | 0.029 | -0.076 | 0.034 |
| L_1034 | Inv_32_2 | Inv_53_2 | Chr_08 | Chr_13 | 7.256 | 0.127 | 0.047 | 36.866 | -1.986 | -1.827 | 0.048 | -0.072 | 0.067 |
| L_1034 | Inv_35_1 | Inv_40_1 | Chr_08 | Chr_10 | 4.784 | 0.320 | 0.035 | 23.161 | 2.111 | 0.695 | 0.049 | 0.080 | 0.024 |
| L_1034 | Inv_35_1 | Inv_58_1 | Chr_08 | Chr_14 | 10.190 | 0.035 | 0.005 | 19.331 | -2.780 | -0.919 | 0.009 | -0.093 | 0.009 |
| L_1034 | Inv_35_1 | Inv_58_2 | Chr_08 | Chr_14 | 10.190 | 0.035 | 0.004 | 26.779 | 2.869 | 1.090 | 0.006 | 0.105 | 0.004 |
| L_1034 | Inv_38_2 | Inv_49_1 | Chr_09 | Chr_12 | 14.306 | 0.006 | 0.002 | 22.082 | -3.164 | -1.165 | 0.002 | -0.095 | 0.004 |
| L_1034 | Inv_38_2 | Inv_49_2 | Chr_09 | Chr_12 | 14.306 | 0.006 | 0.001 | 44.276 | 3.256 | 1.385 | 0.003 | 0.140 | 0.001 |
| L_1034 | Inv_39_1 | Inv_53_2 | Chr_10 | Chr_13 | 10.387 | 0.033 | 0.048 | 18.316 | -1.981 | -1.034 | 0.059 | -0.057 | 0.059 |
| L_1034 | Inv_39_1 | Inv_7_1 | Chr_10 | Chr_02 | 13.028 | 0.012 | 0.035 | 8.891 | -2.107 | -0.694 | 0.048 | -0.071 | 0.047 |
| L_1034 | Inv_39_2 | Inv_55_1 | Chr_10 | Chr_13 | 9.219 | 0.051 | 0.019 | 16.193 | 2.351 | 0.969 | 0.023 | 0.074 | 0.016 |
| L_1034 | Inv_40_1 | Inv_7_1 | Chr_10 | Chr_02 | 8.733 | 0.067 | 0.013 | 17.381 | -2.471 | -0.813 | 0.015 | -0.083 | 0.020 |
| L_1034 | Inv_42_1 | Inv_49_1 | Chr_10 | Chr_12 | 8.069 | 0.089 | 0.058 | 9.811 | 1.897 | 0.624 | 0.072 | 0.070 | 0.049 |
| L_1034 | Inv_42_2 | Inv_49_2 | Chr_10 | Chr_12 | 8.069 | 0.089 | 0.051 | 31.139 | 1.954 | 0.901 | 0.063 | 0.084 | 0.040 |
| L_1034 | Inv_43_1 | Inv_60_1 | Chr_10 | Chr_14 | 7.008 | 0.137 | 0.029 | 15.025 | 2.184 | 0.717 | 0.034 | 0.082 | 0.023 |
| L_1034 | Inv_43_2 | Inv_60_1 | Chr_10 | Chr_14 | 7.008 | 0.137 | 0.023 | 29.760 | -2.267 | -1.013 | 0.031 | -0.064 | 0.031 |
| L_1034 | Inv_49_1 | Inv_58_2 | Chr_12 | Chr_14 | 7.264 | 0.125 | 0.009 | 29.525 | -2.619 | -0.949 | 0.011 | -0.080 | 0.016 |
| L_1034 | Inv_49_1 | Inv_9_1 | Chr_12 | Chr_02 | 6.200 | 0.188 | 0.023 | 17.527 | -2.268 | -0.750 | 0.033 | -0.075 | 0.031 |
| L_1034 | Inv_49_2 | Inv_55_1 | Chr_12 | Chr_13 | 4.542 | 0.318 | 0.038 | 29.009 | 2.072 | 1.057 | 0.045 | 0.054 | 0.032 |
| L_1192 | Inv_17_1 | Inv_25_2 | Chr_04 | Chr_06 | 7.294 | 0.118 | 0.022 | 29.512 | -2.294 | -0.875 | 0.028 | -0.073 | 0.031 |
| L_1192 | Inv_17_2 | Inv_27_1 | Chr_04 | Chr_07 | 9.161 | 0.057 | 0.024 | 19.196 | -2.261 | -0.883 | 0.030 | -0.060 | 0.036 |
| L_1192 | Inv_17_2 | Inv_27_2 | Chr_04 | Chr_07 | 9.161 | 0.057 | 0.011 | 45.232 | 2.556 | 1.070 | 0.015 | 0.109 | 0.009 |
| L_1192 | Inv_17_2 | Inv_32_2 | Chr_04 | Chr_08 | 10.511 | 0.034 | 0.003 | 52.725 | 3.003 | 1.213 | 0.005 | 0.129 | 0.002 |
| L_1192 | Inv_17_2 | Inv_54_1 | Chr_04 | Chr_13 | 5.508 | 0.247 | 0.047 | 31.010 | -1.987 | -0.812 | 0.052 | -0.061 | 0.059 |
| L_1192 | Inv_17_2 | Inv_61_2 | Chr_04 | Chr_14 | 4.417 | 0.358 | 0.043 | 54.922 | -2.028 | -1.102 | 0.047 | -0.072 | 0.059 |
| L_1192 | Inv_19_1 | Inv_25_2 | Chr_04 | Chr_06 | 14.050 | 0.007 | 0.005 | 22.872 | -2.835 | -1.094 | 0.006 | -0.088 | 0.010 |
| L_1192 | Inv_19_1 | Inv_40_1 | Chr_04 | Chr_10 | 5.143 | 0.272 | 0.029 | 25.254 | 2.185 | 0.691 | 0.040 | 0.082 | 0.019 |
| L_1192 | Inv_19_1 | Inv_7_1 | Chr_04 | Chr_02 | 9.088 | 0.058 | 0.005 | 22.577 | -2.815 | -0.895 | 0.007 | -0.092 | 0.010 |
| L_1192 | Inv_19_2 | Inv_32_2 | Chr_04 | Chr_08 | 5.048 | 0.284 | 0.033 | 57.282 | 2.137 | 0.916 | 0.049 | 0.088 | 0.024 |
| L_1192 | Inv_19_2 | Inv_61_2 | Chr_04 | Chr_14 | 4.785 | 0.319 | 0.035 | 56.748 | -2.113 | -1.273 | 0.037 | -0.074 | 0.052 |
| L_1192 | Inv_19_2 | Inv_62_2 | Chr_04 | Chr_14 | 5.719 | 0.223 | 0.017 | 62.150 | -2.388 | -1.642 | 0.016 | -0.084 | 0.030 |
| L_1192 | Inv_23_1 | Inv_61_2 | Chr_05 | Chr_14 | 9.604 | 0.048 | 0.007 | 27.084 | 2.683 | 0.997 | 0.012 | 0.094 | 0.005 |
| L_1192 | Inv_23_2 | Inv_25_2 | Chr_05 | Chr_06 | 5.230 | 0.267 | 0.038 | 44.957 | -2.079 | -0.974 | 0.043 | -0.074 | 0.052 |
| L_1192 | Inv_25_1 | Inv_53_1 | Chr_06 | Chr_13 | 6.272 | 0.183 | 0.060 | 13.231 | 1.880 | 0.592 | 0.083 | 0.068 | 0.043 |
| L_1192 | Inv_25_1 | Inv_54_1 | Chr_06 | Chr_13 | 8.250 | 0.084 | 0.039 | 13.854 | 2.064 | 0.653 | 0.042 | 0.077 | 0.030 |
| L_1192 | Inv_25_2 | Inv_53_2 | Chr_06 | Chr_13 | 6.272 | 0.183 | 0.063 | 31.625 | 1.862 | 0.732 | 0.087 | 0.077 | 0.049 |
| L_1192 | Inv_25_2 | Inv_54_2 | Chr_06 | Chr_13 | 8.250 | 0.084 | 0.014 | 38.657 | 2.453 | 0.914 | 0.017 | 0.102 | 0.011 |
| L_1192 | Inv_27_2 | Inv_29_1 | Chr_07 | Chr_08 | 6.679 | 0.158 | 0.039 | 25.142 | 2.067 | 0.828 | 0.051 | 0.067 | 0.030 |
| L_1192 | Inv_27_2 | Inv_32_2 | Chr_07 | Chr_08 | 6.567 | 0.156 | 0.038 | 40.240 | 2.074 | 0.860 | 0.061 | 0.086 | 0.029 |
| L_1192 | Inv_27_2 | Inv_40_1 | Chr_07 | Chr_10 | 4.592 | 0.338 | 0.055 | 32.593 | 1.915 | 0.757 | 0.078 | 0.063 | 0.044 |
| L_1192 | Inv_28_1 | Inv_43_2 | Chr_08 | Chr_10 | 8.065 | 0.091 | 0.009 | 30.060 | 2.617 | 1.117 | 0.011 | 0.080 | 0.007 |
| L_1192 | Inv_28_2 | Inv_54_1 | Chr_08 | Chr_13 | 11.002 | 0.026 | 0.012 | 24.493 | 2.506 | 0.979 | 0.019 | 0.088 | 0.009 |
| L_1192 | Inv_28_2 | Inv_61_2 | Chr_08 | Chr_14 | 7.030 | 0.137 | 0.034 | 37.409 | -2.120 | -1.147 | 0.046 | -0.076 | 0.047 |
| L_1192 | Inv_29_2 | Inv_54_1 | Chr_08 | Chr_13 | 6.276 | 0.181 | 0.022 | 34.218 | 2.282 | 0.843 | 0.027 | 0.083 | 0.017 |
| L_1192 | Inv_32_1 | Inv_43_2 | Chr_08 | Chr_10 | 5.838 | 0.216 | 0.031 | 29.775 | -2.157 | -0.851 | 0.034 | -0.060 | 0.043 |
| L_1192 | Inv_32_2 | Inv_43_2 | Chr_08 | Chr_10 | 5.838 | 0.216 | 0.038 | 45.264 | 2.074 | 0.860 | 0.061 | 0.086 | 0.028 |
| L_1192 | Inv_43_2 | Inv_62_1 | Chr_10 | Chr_14 | 5.347 | 0.255 | 0.032 | 34.286 | -2.146 | -0.859 | 0.050 | -0.062 | 0.042 |
| L_1192 | Inv_53_1 | Inv_61_2 | Chr_13 | Chr_14 | 9.378 | 0.054 | 0.012 | 23.961 | -2.526 | -0.918 | 0.013 | -0.076 | 0.019 |
| L_1192 | Inv_53_2 | Inv_61_2 | Chr_13 | Chr_14 | 9.378 | 0.054 | 0.040 | 25.205 | 2.050 | 0.793 | 0.058 | 0.085 | 0.029 |
| L_155 | Inv_10b_1 | Inv_15_2 | Chr_02 | Chr_03 | 8.129 | 0.084 | 0.024 | 21.567 | 2.250 | 0.750 | 0.033 | 0.073 | 0.019 |
| L_155 | Inv_10b_1 | Inv_16_2 | Chr_02 | Chr_03 | 8.845 | 0.062 | 0.017 | 22.242 | 2.392 | 0.794 | 0.023 | 0.078 | 0.011 |
| L_155 | Inv_10b_1 | Inv_49_1 | Chr_02 | Chr_12 | 8.331 | 0.077 | 0.012 | 19.165 | 2.517 | 0.741 | 0.013 | 0.087 | 0.008 |
| L_155 | Inv_10b_1 | Inv_51_1 | Chr_02 | Chr_12 | 6.471 | 0.170 | 0.025 | 19.935 | 2.240 | 0.659 | 0.029 | 0.077 | 0.019 |
| L_155 | Inv_10b_1 | Inv_62_2 | Chr_02 | Chr_14 | 4.965 | 0.291 | 0.050 | 27.230 | 1.961 | 0.659 | 0.069 | 0.062 | 0.041 |
| L_155 | Inv_10b_2 | Inv_15_1 | Chr_02 | Chr_03 | 8.129 | 0.084 | 0.056 | 17.308 | 1.910 | 0.672 | 0.082 | 0.059 | 0.046 |
| L_155 | Inv_10b_2 | Inv_16_1 | Chr_02 | Chr_03 | 8.845 | 0.062 | 0.032 | 20.788 | 2.149 | 0.755 | 0.038 | 0.068 | 0.025 |
| L_155 | Inv_10b_2 | Inv_16_2 | Chr_02 | Chr_03 | 8.845 | 0.062 | 0.048 | 24.472 | -1.977 | -0.867 | 0.055 | -0.066 | 0.061 |
| L_155 | Inv_10b_2 | Inv_25_1 | Chr_02 | Chr_06 | 6.804 | 0.143 | 0.016 | 30.282 | 2.403 | 0.873 | 0.023 | 0.072 | 0.011 |
| L_155 | Inv_10b_2 | Inv_29_1 | Chr_02 | Chr_08 | 8.443 | 0.073 | 0.045 | 15.590 | -2.003 | -0.693 | 0.054 | -0.052 | 0.057 |
| L_155 | Inv_10b_2 | Inv_33_1 | Chr_02 | Chr_08 | 7.458 | 0.110 | 0.034 | 21.606 | -2.125 | -0.741 | 0.038 | -0.057 | 0.045 |
| L_155 | Inv_15_1 | Inv_29_1 | Chr_03 | Chr_08 | 5.136 | 0.277 | 0.039 | 17.668 | -2.065 | -0.611 | 0.041 | -0.062 | 0.052 |
| L_155 | Inv_15_1 | Inv_51_2 | Chr_03 | Chr_12 | 8.937 | 0.065 | 0.037 | 17.192 | 2.084 | 0.674 | 0.054 | 0.069 | 0.032 |
| L_155 | Inv_15_1 | Inv_58_1 | Chr_03 | Chr_14 | 5.155 | 0.273 | 0.040 | 18.281 | -2.054 | -0.604 | 0.042 | -0.062 | 0.052 |
| L_155 | Inv_15_1 | Inv_9_2 | Chr_03 | Chr_02 | 8.129 | 0.083 | 0.056 | 17.308 | 1.910 | 0.672 | 0.082 | 0.059 | 0.046 |
| L_155 | Inv_15_2 | Inv_9_1 | Chr_03 | Chr_02 | 8.129 | 0.083 | 0.024 | 21.567 | 2.250 | 0.750 | 0.033 | 0.073 | 0.022 |
| L_155 | Inv_16_1 | Inv_51_2 | Chr_03 | Chr_12 | 10.502 | 0.031 | 0.041 | 14.549 | 2.046 | 0.659 | 0.054 | 0.069 | 0.031 |
| L_155 | Inv_16_1 | Inv_58_1 | Chr_03 | Chr_14 | 7.553 | 0.111 | 0.031 | 14.171 | -2.155 | -0.634 | 0.040 | -0.065 | 0.039 |
| L_155 | Inv_16_1 | Inv_9_2 | Chr_03 | Chr_02 | 8.845 | 0.063 | 0.032 | 20.788 | 2.149 | 0.755 | 0.038 | 0.068 | 0.023 |
| L_155 | Inv_16_2 | Inv_9_1 | Chr_03 | Chr_02 | 8.845 | 0.063 | 0.017 | 22.242 | 2.392 | 0.794 | 0.023 | 0.078 | 0.012 |
| L_155 | Inv_16_2 | Inv_9_2 | Chr_03 | Chr_02 | 8.845 | 0.063 | 0.048 | 24.472 | -1.977 | -0.867 | 0.055 | -0.066 | 0.063 |
| L_155 | Inv_25_1 | Inv_9_2 | Chr_06 | Chr_02 | 6.804 | 0.145 | 0.016 | 30.282 | 2.403 | 0.873 | 0.023 | 0.072 | 0.011 |
| L_155 | Inv_29_1 | Inv_9_2 | Chr_08 | Chr_02 | 8.443 | 0.077 | 0.045 | 15.590 | -2.003 | -0.693 | 0.054 | -0.052 | 0.056 |
| L_155 | Inv_32_2 | Inv_53_2 | Chr_08 | Chr_13 | 20.338 | 0.000 | 0.033 | 16.085 | -2.132 | -10.000 | 0.026 | -0.062 | 0.046 |
| L_155 | Inv_33_1 | Inv_9_2 | Chr_08 | Chr_02 | 7.458 | 0.119 | 0.034 | 21.606 | -2.125 | -0.741 | 0.038 | -0.057 | 0.044 |
| L_155 | Inv_33_2 | Inv_53_1 | Chr_08 | Chr_13 | 20.364 | 0.001 | 0.046 | 5.658 | 1.996 | 0.758 | 0.052 | 0.053 | 0.040 |
| L_155 | Inv_33_2 | Inv_53_2 | Chr_08 | Chr_13 | 20.364 | 0.001 | 0.026 | 17.240 | -2.232 | -10.000 | 0.024 | -0.063 | 0.036 |
| L_155 | Inv_33_2 | Inv_58_1 | Chr_08 | Chr_14 | 7.545 | 0.106 | 0.050 | 17.442 | 1.961 | 0.703 | 0.058 | 0.057 | 0.040 |
| L_155 | Inv_35_1 | Inv_7_1 | Chr_08 | Chr_02 | 9.018 | 0.064 | 0.023 | 13.537 | 2.274 | 0.689 | 0.026 | 0.074 | 0.019 |
| L_155 | Inv_35_1 | Inv_7_2 | Chr_08 | Chr_02 | 9.018 | 0.064 | 0.016 | 18.171 | -2.399 | -0.756 | 0.025 | -0.067 | 0.022 |
| L_155 | Inv_35_2 | Inv_53_2 | Chr_08 | Chr_13 | 7.783 | 0.097 | 0.049 | 36.906 | -1.965 | -10.000 | 0.047 | -0.060 | 0.062 |
| L_155 | Inv_35_2 | Inv_58_1 | Chr_08 | Chr_14 | 10.743 | 0.029 | 0.008 | 23.463 | 2.642 | 1.067 | 0.009 | 0.073 | 0.005 |
| L_155 | Inv_35_2 | Inv_7_1 | Chr_08 | Chr_02 | 9.018 | 0.064 | 0.039 | 21.709 | -2.063 | -0.820 | 0.042 | -0.059 | 0.054 |
| L_155 | Inv_4_2 | Inv_40_1 | Chr_01 | Chr_10 | 6.090 | 0.196 | 0.023 | 26.032 | -2.279 | -0.703 | 0.030 | -0.068 | 0.031 |
| L_155 | Inv_40_2 | Inv_7_1 | Chr_10 | Chr_02 | 4.600 | 0.334 | 0.050 | 35.493 | 1.956 | 0.657 | 0.062 | 0.067 | 0.040 |
| L_155 | Inv_49_1 | Inv_53_1 | Chr_12 | Chr_13 | 9.706 | 0.047 | 0.022 | 10.777 | 2.293 | 0.699 | 0.025 | 0.073 | 0.016 |
| L_155 | Inv_49_1 | Inv_7_1 | Chr_12 | Chr_02 | 4.466 | 0.353 | 0.050 | 25.857 | 1.963 | 0.579 | 0.056 | 0.069 | 0.041 |
| L_155 | Inv_49_1 | Inv_9_1 | Chr_12 | Chr_02 | 8.331 | 0.086 | 0.012 | 19.165 | 2.517 | 0.741 | 0.013 | 0.087 | 0.008 |
| L_155 | Inv_4_1 | Inv_49_2 | Chr_01 | Chr_12 | 5.348 | 0.253 | 0.049 | 26.509 | 1.966 | 0.641 | 0.054 | 0.065 | 0.038 |
| L_155 | Inv_4_2 | Inv_51_1 | Chr_01 | Chr_12 | 5.113 | 0.282 | 0.049 | 26.906 | 1.970 | 0.607 | 0.064 | 0.069 | 0.040 |
| L_155 | Inv_51_1 | Inv_53_1 | Chr_12 | Chr_13 | 12.543 | 0.011 | 0.003 | 14.305 | 2.974 | 0.919 | 0.004 | 0.096 | 0.003 |
| L_155 | Inv_51_1 | Inv_7_1 | Chr_12 | Chr_02 | 5.726 | 0.221 | 0.026 | 26.317 | 2.220 | 0.656 | 0.028 | 0.079 | 0.020 |
| L_155 | Inv_51_1 | Inv_7_2 | Chr_12 | Chr_02 | 5.726 | 0.221 | 0.047 | 25.411 | -1.987 | -0.636 | 0.059 | -0.062 | 0.063 |
| L_155 | Inv_51_1 | Inv_9_1 | Chr_12 | Chr_02 | 6.471 | 0.164 | 0.025 | 19.935 | 2.240 | 0.659 | 0.029 | 0.077 | 0.017 |
| L_155 | Inv_53_1 | Inv_58_1 | Chr_13 | Chr_14 | 12.975 | 0.012 | 0.058 | 4.691 | 1.899 | 0.572 | 0.070 | 0.059 | 0.048 |
| L_155 | Inv_53_2 | Inv_58_1 | Chr_13 | Chr_14 | 12.975 | 0.012 | 0.004 | 26.069 | -2.868 | -1.716 | 0.006 | -0.049 | 0.007 |
| L_155 | Inv_53_2 | Inv_7_1 | Chr_13 | Chr_02 | 4.761 | 0.316 | 0.055 | 40.662 | 1.923 | 1.050 | 0.062 | 0.044 | 0.048 |
| L_155 | Inv_62_2 | Inv_9_1 | Chr_14 | Chr_02 | 4.965 | 0.299 | 0.050 | 27.230 | 1.961 | 0.659 | 0.069 | 0.062 | 0.037 |
| L_444 | Inv_1_1 | Inv_10b_1 | Chr_01 | Chr_02 | 7.760 | 0.103 | 0.041 | 13.032 | 2.042 | 0.689 | 0.046 | 0.077 | 0.033 |
| L_444 | Inv_1_1 | Inv_34_2 | Chr_01 | Chr_08 | 6.132 | 0.195 | 0.043 | 24.338 | -2.025 | -0.717 | 0.056 | -0.073 | 0.061 |
| L_444 | Inv_1_1 | Inv_35_1 | Chr_01 | Chr_08 | 12.138 | 0.017 | 0.028 | 10.822 | 2.194 | 0.732 | 0.032 | 0.086 | 0.020 |
| L_444 | Inv_1_1 | Inv_35_2 | Chr_01 | Chr_08 | 12.138 | 0.017 | 0.018 | 18.557 | -2.368 | -0.910 | 0.027 | -0.082 | 0.031 |
| L_444 | Inv_1_1 | Inv_7_1 | Chr_01 | Chr_02 | 7.155 | 0.123 | 0.029 | 16.436 | 2.185 | 0.737 | 0.032 | 0.083 | 0.024 |
| L_444 | Inv_1_2 | Inv_10b_1 | Chr_01 | Chr_02 | 7.760 | 0.103 | 0.013 | 25.771 | -2.494 | -0.941 | 0.014 | -0.075 | 0.019 |
| L_444 | Inv_1_2 | Inv_10b_2 | Chr_01 | Chr_02 | 7.760 | 0.103 | 0.050 | 30.800 | 1.959 | 0.889 | 0.072 | 0.082 | 0.036 |
| L_444 | Inv_10b_1 | Inv_16_1 | Chr_02 | Chr_03 | 7.481 | 0.111 | 0.026 | 13.741 | -2.226 | -0.745 | 0.031 | -0.074 | 0.035 |
| L_444 | Inv_10b_1 | Inv_16_2 | Chr_02 | Chr_03 | 7.481 | 0.111 | 0.017 | 25.327 | 2.396 | 1.001 | 0.019 | 0.079 | 0.010 |
| L_444 | Inv_10b_1 | Inv_25_1 | Chr_02 | Chr_06 | 6.888 | 0.151 | 0.023 | 18.535 | -2.267 | -0.760 | 0.029 | -0.076 | 0.034 |
| L_444 | Inv_10b_1 | Inv_26_1 | Chr_02 | Chr_06 | 7.425 | 0.118 | 0.023 | 17.195 | -2.267 | -0.760 | 0.029 | -0.076 | 0.033 |
| L_62 | Inv_10b_1 | Inv_29_2 | Chr_02 | Chr_08 | 5.720 | 0.224 | 0.029 | 35.492 | -2.178 | -1.179 | 0.044 | -0.090 | 0.043 |
| L_62 | Inv_10b_1 | Inv_5_2 | Chr_02 | Chr_01 | 5.026 | 0.296 | 0.033 | 36.130 | -2.137 | -1.048 | 0.038 | -0.092 | 0.048 |
| L_62 | Inv_10b_1 | Inv_62_2 | Chr_02 | Chr_14 | 9.820 | 0.039 | 0.047 | 17.494 | -1.990 | -1.087 | 0.074 | -0.082 | 0.063 |
| L_62 | Inv_10b_2 | Inv_23_1 | Chr_02 | Chr_05 | 5.090 | 0.285 | 0.033 | 31.467 | 2.129 | 1.053 | 0.056 | 0.099 | 0.022 |
| L_444 | Inv_10b_2 | Inv_43_2 | Chr_02 | Chr_10 | 6.501 | 0.165 | 0.039 | 41.021 | 2.060 | 0.935 | 0.068 | 0.087 | 0.030 |
| L_444 | Inv_10b_2 | Inv_45_2 | Chr_02 | Chr_10 | 7.280 | 0.120 | 0.051 | 34.170 | 1.955 | 0.909 | 0.063 | 0.084 | 0.040 |
| L_444 | Inv_13_2 | Inv_4_2 | Chr_03 | Chr_01 | 7.521 | 0.113 | 0.016 | 43.150 | 2.413 | 0.963 | 0.028 | 0.106 | 0.011 |
| L_444 | Inv_13_2 | Inv_6b_2 | Chr_03 | Chr_01 | 6.312 | 0.177 | 0.023 | 48.463 | 2.281 | 0.944 | 0.037 | 0.100 | 0.016 |
| L_62 | Inv_14_1 | Inv_32_1 | Chr_03 | Chr_08 | 6.853 | 0.147 | 0.033 | 18.799 | -2.131 | -0.900 | 0.038 | -0.093 | 0.049 |
| L_62 | Inv_14_1 | Inv_40_2 | Chr_03 | Chr_10 | 7.341 | 0.115 | 0.047 | 22.176 | -1.984 | -1.027 | 0.054 | -0.083 | 0.065 |
| L_62 | Inv_14_2 | Inv_32_1 | Chr_03 | Chr_08 | 6.853 | 0.147 | 0.040 | 21.024 | 2.058 | 0.906 | 0.050 | 0.105 | 0.029 |
| L_62 | Inv_14_2 | Inv_40_2 | Chr_03 | Chr_10 | 7.341 | 0.115 | 0.026 | 33.653 | 2.231 | 1.085 | 0.039 | 0.121 | 0.019 |
| L_444 | Inv_16_1 | Inv_4_1 | Chr_03 | Chr_01 | 8.217 | 0.087 | 0.009 | 21.149 | -2.605 | -0.866 | 0.013 | -0.089 | 0.016 |
| L_444 | Inv_16_1 | Inv_4_2 | Chr_03 | Chr_01 | 8.217 | 0.087 | 0.055 | 15.746 | 1.923 | 0.725 | 0.065 | 0.069 | 0.045 |
| L_444 | Inv_16_1 | Inv_6b_1 | Chr_03 | Chr_01 | 7.237 | 0.125 | 0.018 | 16.725 | -2.361 | -0.787 | 0.021 | -0.079 | 0.029 |
| L_444 | Inv_16_2 | Inv_4_1 | Chr_03 | Chr_01 | 8.217 | 0.087 | 0.028 | 24.082 | 2.204 | 0.860 | 0.034 | 0.082 | 0.019 |
| L_444 | Inv_16_2 | Inv_49_1 | Chr_03 | Chr_12 | 9.090 | 0.012 | 0.004 | 43.581 | -2.889 | -1.336 | 0.005 | -0.100 | 0.009 |
| L_62 | Inv_18_2 | Inv_57_2 | Chr_04 | Chr_14 | 6.051 | 0.200 | 0.020 | 52.886 | -2.335 | -2.108 | 0.020 | -0.108 | 0.034 |
| L_62 | Inv_18_2 | Inv_59_1 | Chr_04 | Chr_14 | 8.934 | 0.062 | 0.016 | 26.173 | -2.418 | -1.308 | 0.022 | -0.094 | 0.029 |
| L_62 | Inv_18_2 | Inv_62_1 | Chr_04 | Chr_14 | 8.469 | 0.076 | 0.008 | 31.458 | -2.638 | -1.419 | 0.011 | -0.099 | 0.018 |
| L_62 | Inv_18_2 | Inv_62_2 | Chr_04 | Chr_14 | 8.469 | 0.076 | 0.025 | 36.223 | 2.238 | 1.188 | 0.034 | 0.128 | 0.018 |
| L_444 | Inv_22_1 | Inv_35_2 | Chr_05 | Chr_08 | 7.323 | 0.119 | 0.017 | 27.311 | -2.396 | -0.886 | 0.019 | -0.078 | 0.028 |
| L_444 | Inv_22_2 | Inv_35_2 | Chr_05 | Chr_08 | 7.323 | 0.119 | 0.029 | 34.457 | 2.177 | 0.848 | 0.037 | 0.094 | 0.021 |
| L_444 | Inv_22_2 | Inv_58_1 | Chr_05 | Chr_14 | 5.531 | 0.238 | 0.049 | 25.011 | 1.970 | 0.758 | 0.060 | 0.070 | 0.039 |
| L_444 | Inv_22_2 | Inv_7_2 | Chr_05 | Chr_02 | 6.923 | 0.144 | 0.044 | 34.631 | 2.011 | 0.852 | 0.060 | 0.087 | 0.034 |
| L_62 | Inv_23_1 | Inv_29_1 | Chr_05 | Chr_08 | 7.816 | 0.107 | 0.012 | 19.191 | -2.505 | -1.058 | 0.021 | -0.106 | 0.023 |
| L_62 | Inv_23_1 | Inv_29_2 | Chr_05 | Chr_08 | 7.816 | 0.107 | 0.044 | 19.092 | 2.011 | 1.046 | 0.051 | 0.089 | 0.031 |
| L_62 | Inv_23_1 | Inv_8_2 | Chr_05 | Chr_02 | 5.871 | 0.220 | 0.063 | 20.443 | 1.856 | 0.893 | 0.100 | 0.086 | 0.050 |
| L_62 | Inv_23_2 | Inv_53_2 | Chr_05 | Chr_13 | 5.136 | 0.277 | 0.061 | 36.697 | 1.872 | 0.924 | 0.107 | 0.103 | 0.043 |
| L_62 | Inv_23_2 | Inv_7_2 | Chr_05 | Chr_02 | 7.838 | 0.096 | 0.030 | 33.284 | -2.171 | -1.566 | 0.031 | -0.100 | 0.053 |
| L_62 | Inv_23_2 | Inv_8_2 | Chr_05 | Chr_02 | 5.871 | 0.220 | 0.034 | 39.844 | -2.116 | -1.331 | 0.039 | -0.098 | 0.055 |
| L_444 | Inv_25_1 | Inv_29_2 | Chr_06 | Chr_08 | 5.384 | 0.249 | 0.030 | 35.902 | -2.168 | -0.835 | 0.042 | -0.076 | 0.044 |
| L_444 | Inv_25_1 | Inv_7_1 | Chr_06 | Chr_02 | 10.188 | 0.035 | 0.014 | 14.931 | -2.456 | -0.823 | 0.019 | -0.082 | 0.021 |
| L_444 | Inv_25_2 | Inv_53_2 | Chr_06 | Chr_13 | 8.378 | 0.083 | 0.023 | 37.146 | 2.272 | 0.969 | 0.031 | 0.099 | 0.017 |
| L_444 | Inv_26_1 | Inv_29_1 | Chr_06 | Chr_08 | 7.135 | 0.133 | 0.060 | 13.822 | 1.879 | 0.627 | 0.069 | 0.073 | 0.050 |
| L_444 | Inv_26_1 | Inv_29_2 | Chr_06 | Chr_08 | 7.135 | 0.133 | 0.011 | 37.088 | -2.536 | -0.992 | 0.016 | -0.088 | 0.020 |
| L_444 | Inv_26_1 | Inv_7_1 | Chr_06 | Chr_02 | 11.749 | 0.016 | 0.005 | 16.720 | -2.791 | -0.939 | 0.007 | -0.092 | 0.010 |
| L_444 | Inv_26_2 | Inv_29_2 | Chr_06 | Chr_08 | 7.135 | 0.133 | 0.058 | 27.029 | 1.895 | 0.746 | 0.091 | 0.081 | 0.048 |
| L_444 | Inv_26_2 | Inv_40_1 | Chr_06 | Chr_10 | 5.116 | 0.283 | 0.046 | 30.354 | 1.995 | 0.763 | 0.058 | 0.074 | 0.035 |
| L_444 | Inv_26_2 | Inv_53_2 | Chr_06 | Chr_13 | 8.750 | 0.065 | 0.023 | 35.567 | 2.272 | 0.969 | 0.031 | 0.099 | 0.016 |
| L_62 | Inv_29_1 | Inv_37_2 | Chr_08 | Chr_09 | 11.102 | 0.024 | 0.043 | 12.579 | 2.020 | 0.919 | 0.048 | 0.100 | 0.034 |
| L_444 | Inv_29_1 | Inv_58_1 | Chr_08 | Chr_14 | 9.099 | 0.060 | 0.014 | 15.466 | 2.456 | 0.814 | 0.021 | 0.095 | 0.009 |
| L_444 | Inv_29_1 | Inv_58_2 | Chr_08 | Chr_14 | 9.099 | 0.060 | 0.043 | 17.661 | -2.027 | -0.855 | 0.061 | -0.060 | 0.058 |
| L_502 | Inv_21_1 | Inv_32_1 | Chr_05 | Chr_08 | 8.171 | 0.085 | 0.031 | 15.125 | 2.161 | 0.684 | 0.041 | 0.080 | 0.022 |
| L_502 | Inv_21_1 | Inv_32_2 | Chr_05 | Chr_08 | 8.171 | 0.085 | 0.043 | 18.013 | -2.023 | -0.730 | 0.050 | -0.062 | 0.056 |
| L_502 | Inv_21_2 | Inv_29_1 | Chr_05 | Chr_08 | 6.699 | 0.152 | 0.018 | 31.187 | 2.357 | 0.842 | 0.022 | 0.085 | 0.014 |
| L_502 | Inv_21_2 | Inv_31_2 | Chr_05 | Chr_08 | 5.856 | 0.213 | 0.045 | 37.542 | 2.006 | 0.760 | 0.066 | 0.082 | 0.034 |
| L_502 | Inv_21_2 | Inv_32_1 | Chr_05 | Chr_08 | 8.171 | 0.085 | 0.013 | 29.812 | -2.472 | -0.914 | 0.014 | -0.080 | 0.021 |
| L_502 | Inv_21_2 | Inv_32_2 | Chr_05 | Chr_08 | 8.171 | 0.085 | 0.022 | 34.614 | 2.284 | 0.856 | 0.027 | 0.095 | 0.015 |
| L_502 | Inv_21_2 | Inv_54_1 | Chr_05 | Chr_13 | 7.792 | 0.097 | 0.018 | 26.814 | 2.357 | 0.842 | 0.022 | 0.085 | 0.013 |
| L_502 | Inv_21_2 | Inv_55_1 | Chr_05 | Chr_13 | 7.792 | 0.100 | 0.018 | 26.814 | 2.357 | 0.842 | 0.022 | 0.085 | 0.012 |
| L_502 | Inv_22_1 | Inv_31_2 | Chr_05 | Chr_08 | 8.351 | 0.079 | 0.016 | 23.308 | -2.410 | -0.874 | 0.019 | -0.070 | 0.023 |
| L_502 | Inv_22_2 | Inv_47_1 | Chr_05 | Chr_11 | 12.324 | 0.015 | 0.014 | 19.049 | 2.465 | 0.871 | 0.022 | 0.091 | 0.007 |
| L_502 | Inv_22_2 | Inv_48_1 | Chr_05 | Chr_11 | 10.570 | 0.030 | 0.027 | 18.104 | 2.213 | 0.778 | 0.035 | 0.082 | 0.019 |
| L_502 | Inv_22_2 | Inv_49_2 | Chr_05 | Chr_12 | 5.902 | 0.208 | 0.029 | 45.106 | -2.181 | -1.085 | 0.036 | -0.077 | 0.038 |
| L_502 | Inv_22_2 | Inv_54_1 | Chr_05 | Chr_13 | 8.066 | 0.091 | 0.030 | 21.781 | 2.170 | 0.767 | 0.036 | 0.078 | 0.021 |
| L_502 | Inv_22_2 | Inv_55_1 | Chr_05 | Chr_13 | 8.066 | 0.089 | 0.030 | 21.781 | 2.170 | 0.767 | 0.036 | 0.078 | 0.021 |
| L_502 | Inv_27_1 | Inv_31_2 | Chr_07 | Chr_08 | 14.458 | 0.005 | 0.001 | 31.340 | -3.387 | -1.321 | 0.001 | -0.104 | 0.002 |
| L_502 | Inv_27_1 | Inv_32_2 | Chr_07 | Chr_08 | 9.755 | 0.041 | 0.014 | 24.178 | -2.454 | -0.913 | 0.019 | -0.077 | 0.023 |
| L_502 | Inv_27_1 | Inv_54_1 | Chr_07 | Chr_13 | 6.725 | 0.157 | 0.016 | 23.509 | 2.411 | 0.761 | 0.019 | 0.091 | 0.010 |
| L_502 | Inv_27_1 | Inv_55_1 | Chr_07 | Chr_13 | 6.725 | 0.160 | 0.016 | 23.509 | 2.411 | 0.761 | 0.019 | 0.091 | 0.010 |
| L_502 | Inv_27_1 | Inv_61_1 | Chr_07 | Chr_14 | 6.990 | 0.131 | 0.037 | 17.267 | 2.082 | 0.656 | 0.042 | 0.078 | 0.030 |
| L_502 | Inv_27_1 | Inv_61_2 | Chr_07 | Chr_14 | 6.990 | 0.131 | 0.021 | 29.955 | -2.303 | -0.860 | 0.030 | -0.073 | 0.029 |
| L_502 | Inv_27_1 | Inv_9_2 | Chr_07 | Chr_02 | 7.586 | 0.107 | 0.029 | 26.414 | -2.182 | -0.889 | 0.033 | -0.065 | 0.037 |
| L_502 | Inv_29_1 | Inv_49_1 | Chr_08 | Chr_12 | 5.832 | 0.204 | 0.031 | 18.734 | 2.161 | 0.684 | 0.041 | 0.078 | 0.024 |
| L_502 | Inv_29_1 | Inv_4_1 | Chr_08 | Chr_01 | 12.796 | 0.012 | 0.009 | 12.775 | 2.626 | 0.834 | 0.012 | 0.097 | 0.005 |
| L_502 | Inv_29_2 | Inv_4_2 | Chr_08 | Chr_01 | 12.796 | 0.012 | 0.013 | 28.149 | 2.496 | 0.978 | 0.017 | 0.104 | 0.009 |
| L_502 | Inv_31_2 | Inv_54_1 | Chr_08 | Chr_13 | 14.161 | 0.007 | 0.013 | 16.778 | -2.482 | -0.926 | 0.018 | -0.077 | 0.025 |
| L_502 | Inv_31_2 | Inv_55_1 | Chr_08 | Chr_13 | 14.161 | 0.007 | 0.013 | 16.778 | -2.482 | -0.926 | 0.018 | -0.077 | 0.022 |
| L_502 | Inv_31_2 | Inv_61_2 | Chr_08 | Chr_14 | 9.807 | 0.045 | 0.005 | 44.214 | 2.782 | 1.055 | 0.012 | 0.118 | 0.004 |
| L_502 | Inv_31_2 | Inv_9_1 | Chr_08 | Chr_02 | 6.783 | 0.146 | 0.013 | 31.922 | -2.491 | -0.909 | 0.018 | -0.073 | 0.022 |
| L_502 | Inv_32_2 | Inv_61_2 | Chr_08 | Chr_14 | 9.461 | 0.053 | 0.008 | 41.438 | 2.656 | 1.004 | 0.013 | 0.112 | 0.007 |
| L_502 | Inv_35_1 | Inv_4_2 | Chr_08 | Chr_01 | 7.898 | 0.093 | 0.016 | 23.155 | 2.398 | 1.022 | 0.020 | 0.070 | 0.012 |
| L_502 | Inv_35_2 | Inv_43_2 | Chr_08 | Chr_10 | 9.040 | 0.057 | 0.052 | 27.050 | 1.947 | 0.859 | 0.076 | 0.079 | 0.042 |
| L_502 | Inv_35_2 | Inv_45_2 | Chr_08 | Chr_10 | 12.190 | 0.015 | 0.011 | 34.901 | 2.558 | 1.108 | 0.020 | 0.107 | 0.010 |
| L_502 | Inv_40_1 | Inv_61_1 | Chr_10 | Chr_14 | 11.130 | 0.026 | 0.003 | 19.664 | 2.926 | 0.930 | 0.005 | 0.110 | 0.002 |
| L_502 | Inv_43_2 | Inv_61_2 | Chr_10 | Chr_14 | 8.182 | 0.086 | 0.022 | 36.981 | -2.287 | -1.226 | 0.030 | -0.081 | 0.035 |
| L_502 | Inv_45_2 | Inv_61_2 | Chr_10 | Chr_14 | 8.240 | 0.077 | 0.028 | 34.249 | -2.200 | -1.184 | 0.030 | -0.078 | 0.042 |
| L_502 | Inv_47_1 | Inv_62_1 | Chr_11 | Chr_14 | 6.388 | 0.169 | 0.028 | 18.089 | -2.199 | -0.695 | 0.040 | -0.071 | 0.038 |
| L_502 | Inv_47_1 | Inv_62_2 | Chr_11 | Chr_14 | 6.388 | 0.169 | 0.047 | 26.164 | 1.989 | 0.771 | 0.055 | 0.068 | 0.039 |
| L_502 | Inv_49_2 | Inv_61_2 | Chr_12 | Chr_14 | 8.122 | 0.092 | 0.007 | 52.617 | -2.718 | -1.580 | 0.005 | -0.095 | 0.014 |
| L_502 | Inv_52_1 | Inv_62_2 | Chr_12 | Chr_14 | 18.282 | 0.001 | 0.002 | 18.358 | -3.098 | -1.220 | 0.003 | -0.083 | 0.004 |
| L_502 | Inv_52_1 | Inv_9_1 | Chr_12 | Chr_02 | 8.965 | 0.061 | 0.042 | 9.493 | 2.033 | 0.643 | 0.058 | 0.072 | 0.033 |
| L_541 | Inv_10b_1 | Inv_28_1 | Chr_02 | Chr_08 | 9.363 | 0.054 | 0.013 | 17.817 | -2.495 | -0.787 | 0.018 | -0.082 | 0.019 |
| L_541 | Inv_10b_1 | Inv_28_2 | Chr_02 | Chr_08 | 9.363 | 0.054 | 0.015 | 25.657 | 2.439 | 0.879 | 0.019 | 0.089 | 0.008 |
| L_541 | Inv_10b_1 | Inv_29_2 | Chr_02 | Chr_08 | 10.329 | 0.035 | 0.057 | 14.479 | 1.901 | 0.698 | 0.067 | 0.067 | 0.048 |
| L_541 | Inv_10b_2 | Inv_27_1 | Chr_02 | Chr_07 | 8.581 | 0.074 | 0.015 | 26.616 | 2.429 | 0.934 | 0.016 | 0.082 | 0.011 |
| L_541 | Inv_10b_2 | Inv_27_2 | Chr_02 | Chr_07 | 8.581 | 0.074 | 0.017 | 36.569 | -2.393 | -1.180 | 0.023 | -0.084 | 0.028 |
| L_541 | Inv_12_2 | Inv_47_1 | Chr_03 | Chr_11 | 10.970 | 0.029 | 0.038 | 15.261 | -2.075 | -0.766 | 0.047 | -0.065 | 0.052 |
| L_541 | Inv_12_2 | Inv_48_1 | Chr_03 | Chr_11 | 13.532 | 0.008 | 0.024 | 14.996 | -2.258 | -0.843 | 0.030 | -0.071 | 0.034 |
| L_541 | Inv_27_1 | Inv_8_2 | Chr_07 | Chr_02 | 5.821 | 0.213 | 0.036 | 29.693 | 2.096 | 0.817 | 0.039 | 0.069 | 0.029 |
| L_541 | Inv_27_1 | Inv_9_2 | Chr_07 | Chr_02 | 8.998 | 0.060 | 0.008 | 30.252 | 2.641 | 1.034 | 0.014 | 0.089 | 0.006 |
| L_541 | Inv_27_2 | Inv_9_2 | Chr_07 | Chr_02 | 8.998 | 0.060 | 0.022 | 32.331 | -2.296 | -1.137 | 0.023 | -0.080 | 0.033 |
| L_541 | Inv_28_1 | Inv_8_1 | Chr_08 | Chr_02 | 6.840 | 0.143 | 0.029 | 18.643 | -2.181 | -0.686 | 0.041 | -0.072 | 0.041 |
| L_541 | Inv_28_1 | Inv_9_1 | Chr_08 | Chr_02 | 8.915 | 0.065 | 0.019 | 16.244 | -2.338 | -0.736 | 0.028 | -0.077 | 0.027 |
| L_541 | Inv_28_2 | Inv_43_2 | Chr_08 | Chr_10 | 12.366 | 0.013 | 0.003 | 43.309 | 2.936 | 1.232 | 0.007 | 0.121 | 0.003 |
| L_541 | Inv_28_2 | Inv_8_1 | Chr_08 | Chr_02 | 6.840 | 0.143 | 0.038 | 25.540 | 2.080 | 0.746 | 0.048 | 0.075 | 0.026 |
| L_541 | Inv_28_2 | Inv_9_1 | Chr_08 | Chr_02 | 8.915 | 0.065 | 0.019 | 24.623 | 2.345 | 0.845 | 0.021 | 0.085 | 0.013 |
| L_541 | Inv_29_1 | Inv_8_1 | Chr_08 | Chr_02 | 12.126 | 0.018 | 0.033 | 9.546 | -2.132 | -0.671 | 0.041 | -0.070 | 0.044 |
| L_541 | Inv_29_1 | Inv_9_1 | Chr_08 | Chr_02 | 10.512 | 0.030 | 0.047 | 9.416 | -1.982 | -0.622 | 0.060 | -0.065 | 0.064 |
| L_541 | Inv_29_2 | Inv_43_2 | Chr_08 | Chr_10 | 17.217 | 0.002 | 0.000 | 51.965 | 3.749 | 1.561 | 0.001 | 0.161 | 0.000 |
| L_541 | Inv_29_2 | Inv_8_1 | Chr_08 | Chr_02 | 12.126 | 0.018 | 0.023 | 17.571 | 2.270 | 0.837 | 0.028 | 0.081 | 0.017 |
| L_541 | Inv_29_2 | Inv_9_1 | Chr_08 | Chr_02 | 10.512 | 0.030 | 0.029 | 18.490 | 2.180 | 0.804 | 0.042 | 0.077 | 0.023 |
| L_541 | Inv_32_2 | Inv_53_1 | Chr_08 | Chr_13 | 10.563 | 0.032 | 0.003 | 38.337 | -2.989 | -1.378 | 0.004 | -0.087 | 0.008 |
| L_541 | Inv_33_1 | Inv_43_2 | Chr_08 | Chr_10 | 6.722 | 0.150 | 0.057 | 20.906 | 1.905 | 0.845 | 0.061 | 0.054 | 0.046 |
| L_541 | Inv_33_2 | Inv_53_1 | Chr_08 | Chr_13 | 9.055 | 0.064 | 0.007 | 37.017 | -2.699 | -1.263 | 0.007 | -0.078 | 0.013 |
| L_541 | Inv_40_1 | Inv_58_2 | Chr_10 | Chr_14 | 7.394 | 0.121 | 0.022 | 24.963 | 2.293 | 0.839 | 0.032 | 0.078 | 0.015 |
| L_541 | Inv_43_1 | Inv_58_1 | Chr_10 | Chr_14 | 17.516 | 0.001 | 0.021 | 8.486 | 2.312 | 0.727 | 0.028 | 0.087 | 0.015 |
| L_541 | Inv_43_2 | Inv_58_1 | Chr_10 | Chr_14 | 17.516 | 0.001 | 0.005 | 20.077 | -2.817 | -1.318 | 0.006 | -0.078 | 0.010 |
| L_541 | Inv_49_2 | Inv_47_1 | Chr_12 | Chr_11 | 5.442 | 0.246 | 0.035 | 32.560 | 2.109 | 0.789 | 0.042 | 0.073 | 0.027 |
| L_541 | Inv_47_1 | Inv_51_2 | Chr_11 | Chr_12 | 5.361 | 0.257 | 0.035 | 33.051 | 2.109 | 0.789 | 0.042 | 0.073 | 0.024 |
| L_541 | Inv_53_1 | Inv_58_1 | Chr_13 | Chr_14 | 12.814 | 0.011 | 0.009 | 15.588 | -2.605 | -0.830 | 0.012 | -0.086 | 0.017 |
| L_541 | Inv_53_1 | Inv_58_2 | Chr_13 | Chr_14 | 12.814 | 0.011 | 0.005 | 25.187 | 2.808 | 1.006 | 0.007 | 0.106 | 0.003 |
| L_541 | Inv_55_1 | Inv_58_2 | Chr_13 | Chr_14 | 7.273 | 0.128 | 0.039 | 22.997 | 2.067 | 0.737 | 0.050 | 0.074 | 0.033 |
| L_541 | Inv_55_1 | Inv_9_1 | Chr_13 | Chr_02 | 7.337 | 0.118 | 0.036 | 17.059 | -2.097 | -0.661 | 0.042 | -0.070 | 0.048 |
| L_62 | Inv_29_2 | Inv_37_1 | Chr_08 | Chr_09 | 11.102 | 0.024 | 0.028 | 18.297 | 2.201 | 1.110 | 0.045 | 0.106 | 0.022 |
| L_444 | Inv_29_2 | Inv_49_1 | Chr_08 | Chr_12 | 7.496 | 0.023 | 0.008 | 42.415 | 2.672 | 0.978 | 0.009 | 0.113 | 0.006 |
| L_444 | Inv_29_2 | Inv_58_2 | Chr_08 | Chr_14 | 9.099 | 0.060 | 0.028 | 30.388 | 2.191 | 0.918 | 0.038 | 0.093 | 0.021 |
| L_62 | Inv_32_1 | Inv_37_2 | Chr_08 | Chr_09 | 9.882 | 0.044 | 0.064 | 12.447 | 1.856 | 0.836 | 0.075 | 0.093 | 0.046 |
| L_62 | Inv_32_1 | Inv_39_1 | Chr_08 | Chr_10 | 11.589 | 0.020 | 0.013 | 13.336 | -2.489 | -1.051 | 0.021 | -0.106 | 0.020 |
| L_62 | Inv_32_1 | Inv_48_1 | Chr_08 | Chr_11 | 9.950 | 0.036 | 0.033 | 8.024 | 2.131 | 0.962 | 0.047 | 0.094 | 0.027 |
| L_62 | Inv_32_2 | Inv_39_1 | Chr_08 | Chr_10 | 11.589 | 0.020 | 0.004 | 24.128 | 2.899 | 1.398 | 0.006 | 0.144 | 0.001 |
| L_444 | Inv_32_2 | Inv_40_1 | Chr_08 | Chr_10 | 8.290 | 0.083 | 0.009 | 27.983 | 2.608 | 0.912 | 0.010 | 0.105 | 0.005 |
| L_62 | Inv_32_2 | Inv_40_1 | Chr_08 | Chr_10 | 11.609 | 0.019 | 0.010 | 16.233 | 2.561 | 1.286 | 0.012 | 0.116 | 0.005 |
| L_444 | Inv_34_1 | Inv_40_1 | Chr_08 | Chr_10 | 8.833 | 0.065 | 0.021 | 15.931 | -2.310 | -0.765 | 0.023 | -0.080 | 0.031 |
| L_444 | Inv_34_1 | Inv_40_2 | Chr_08 | Chr_10 | 8.833 | 0.065 | 0.057 | 16.421 | 1.903 | 0.781 | 0.070 | 0.066 | 0.046 |
| L_444 | Inv_34_2 | Inv_40_1 | Chr_08 | Chr_10 | 8.833 | 0.065 | 0.015 | 22.581 | 2.431 | 0.844 | 0.017 | 0.097 | 0.011 |
| L_444 | Inv_34_2 | Inv_40_2 | Chr_08 | Chr_10 | 8.833 | 0.065 | 0.013 | 36.081 | -2.493 | -1.243 | 0.012 | -0.087 | 0.026 |
| L_444 | Inv_34_2 | Inv_43_2 | Chr_08 | Chr_10 | 7.257 | 0.124 | 0.047 | 26.500 | -1.988 | -0.862 | 0.050 | -0.072 | 0.062 |
| L_62 | Inv_35_1 | Inv_40_1 | Chr_08 | Chr_10 | 8.664 | 0.067 | 0.041 | 9.542 | 2.046 | 0.870 | 0.057 | 0.097 | 0.030 |
| L_62 | Inv_35_1 | Inv_62_1 | Chr_08 | Chr_14 | 13.712 | 0.005 | 0.062 | 5.964 | 1.870 | 0.782 | 0.094 | 0.091 | 0.047 |
| L_444 | Inv_35_2 | Inv_43_2 | Chr_08 | Chr_10 | 19.215 | 0.001 | 0.006 | 20.941 | -2.737 | -1.449 | 0.006 | -0.100 | 0.017 |
| L_62 | Inv_35_2 | Inv_62_1 | Chr_08 | Chr_14 | 13.712 | 0.005 | 0.000 | 34.490 | -3.644 | -1.883 | 0.000 | -0.141 | 0.002 |
| L_62 | Inv_35_2 | Inv_62_2 | Chr_08 | Chr_14 | 13.712 | 0.005 | 0.043 | 17.057 | 2.026 | 1.039 | 0.086 | 0.113 | 0.031 |
| L_62 | Inv_36_2 | Inv_39_1 | Chr_09 | Chr_10 | 5.549 | 0.236 | 0.035 | 30.363 | 2.107 | 1.154 | 0.042 | 0.091 | 0.028 |
| L_62 | Inv_36_2 | Inv_53_1 | Chr_09 | Chr_13 | 8.401 | 0.077 | 0.009 | 31.072 | 2.623 | 1.498 | 0.010 | 0.115 | 0.005 |
| L_62 | Inv_37_1 | Inv_53_1 | Chr_09 | Chr_13 | 7.887 | 0.100 | 0.040 | 13.966 | -2.059 | -0.865 | 0.059 | -0.089 | 0.054 |
| L_62 | Inv_37_1 | Inv_7_1 | Chr_09 | Chr_02 | 6.065 | 0.205 | 0.043 | 16.824 | -2.028 | -0.853 | 0.059 | -0.087 | 0.058 |
| L_62 | Inv_37_1 | Inv_7_2 | Chr_09 | Chr_02 | 6.065 | 0.205 | 0.028 | 33.493 | 2.201 | 1.110 | 0.045 | 0.106 | 0.017 |
| L_444 | Inv_4_2 | Inv_58_2 | Chr_01 | Chr_14 | 4.265 | 0.383 | 0.052 | 51.825 | 1.941 | 0.824 | 0.064 | 0.082 | 0.041 |
| L_444 | Inv_40_2 | Inv_49_1 | Chr_10 | Chr_12 | 4.125 | 0.139 | 0.049 | 46.238 | -1.970 | -0.899 | 0.061 | -0.068 | 0.070 |
| L_62 | Inv_40_2 | Inv_49_1 | Chr_10 | Chr_12 | 5.098 | 0.288 | 0.036 | 30.704 | 2.098 | 1.084 | 0.050 | 0.093 | 0.027 |
| L_444 | Inv_43_1 | Inv_6b_1 | Chr_10 | Chr_01 | 7.432 | 0.118 | 0.047 | 10.061 | 1.986 | 0.663 | 0.065 | 0.072 | 0.039 |
| L_444 | Inv_43_1 | Inv_6b_2 | Chr_10 | Chr_01 | 7.432 | 0.118 | 0.023 | 22.569 | -2.280 | -0.889 | 0.029 | -0.065 | 0.029 |
| L_444 | Inv_43_2 | Inv_6b_1 | Chr_10 | Chr_01 | 7.432 | 0.118 | 0.045 | 18.313 | -2.002 | -0.761 | 0.057 | -0.062 | 0.056 |
| L_444 | Inv_43_2 | Inv_6b_2 | Chr_10 | Chr_01 | 7.432 | 0.118 | 0.013 | 47.883 | 2.482 | 1.011 | 0.023 | 0.110 | 0.011 |
| L_444 | Inv_45_1 | Inv_6b_2 | Chr_10 | Chr_01 | 6.147 | 0.186 | 0.030 | 23.598 | -2.173 | -0.846 | 0.045 | -0.060 | 0.043 |
| L_444 | Inv_45_2 | Inv_6b_2 | Chr_10 | Chr_01 | 6.147 | 0.186 | 0.038 | 41.902 | 2.074 | 0.875 | 0.058 | 0.091 | 0.026 |
| L_444 | Inv_49_1 | Inv_45_2 | Chr_12 | Chr_10 | 4.992 | 0.091 | 0.025 | 48.289 | -2.234 | -1.008 | 0.027 | -0.078 | 0.036 |
| L_62 | Inv_49_2 | Inv_5_1 | Chr_12 | Chr_01 | 11.000 | 0.025 | 0.025 | 17.134 | -2.244 | -1.171 | 0.041 | -0.084 | 0.039 |
| L_62 | Inv_53_2 | Inv_8_2 | Chr_13 | Chr_02 | 5.823 | 0.215 | 0.021 | 49.631 | -2.300 | -1.644 | 0.029 | -0.106 | 0.043 |
| L_444 | Inv_58_2 | Inv_6b_2 | Chr_14 | Chr_01 | 5.289 | 0.265 | 0.033 | 53.398 | 2.136 | 0.929 | 0.047 | 0.093 | 0.025 |
| L_62 | Inv_59_1 | Inv_7_1 | Chr_14 | Chr_02 | 4.545 | 0.348 | 0.057 | 18.572 | 1.902 | 0.799 | 0.064 | 0.093 | 0.049 |
| L_62 | Inv_62_1 | Inv_7_1 | Chr_14 | Chr_02 | 6.722 | 0.153 | 0.022 | 17.340 | 2.285 | 0.964 | 0.036 | 0.112 | 0.018 |
| L_664 | Inv_10b_1 | Inv_56_1 | Chr_02 | Chr_13 | 7.200 | 0.123 | 0.018 | 18.118 | 2.365 | 0.764 | 0.025 | 0.088 | 0.012 |
| L_664 | Inv_10b_1 | Inv_56_2 | Chr_02 | Chr_13 | 7.200 | 0.123 | 0.049 | 20.630 | -1.966 | -0.734 | 0.066 | -0.063 | 0.058 |
| L_664 | Inv_10b_1 | Inv_62_1 | Chr_02 | Chr_14 | 5.760 | 0.221 | 0.046 | 17.689 | -1.994 | -0.638 | 0.056 | -0.067 | 0.061 |
| L_664 | Inv_10b_2 | Inv_24_2 | Chr_02 | Chr_06 | 8.056 | 0.090 | 0.009 | 53.439 | 2.617 | 1.110 | 0.014 | 0.112 | 0.007 |
| L_664 | Inv_10b_2 | Inv_43_1 | Chr_02 | Chr_10 | 7.016 | 0.133 | 0.059 | 18.484 | 1.889 | 0.738 | 0.086 | 0.062 | 0.047 |
| L_664 | Inv_20_2 | Inv_39_2 | Chr_04 | Chr_10 | 6.065 | 0.200 | 0.040 | 42.504 | 2.058 | 0.857 | 0.061 | 0.087 | 0.028 |
| L_664 | Inv_21_1 | Inv_48_1 | Chr_05 | Chr_11 | 7.679 | 0.105 | 0.049 | 13.481 | -1.965 | -0.632 | 0.055 | -0.066 | 0.063 |
| L_664 | Inv_21_1 | Inv_50_2 | Chr_05 | Chr_12 | 8.517 | 0.075 | 0.014 | 29.002 | 2.456 | 0.896 | 0.018 | 0.094 | 0.008 |
| L_664 | Inv_21_1 | Inv_51_2 | Chr_05 | Chr_12 | 8.517 | 0.074 | 0.014 | 29.002 | 2.456 | 0.896 | 0.018 | 0.094 | 0.009 |
| L_664 | Inv_21_2 | Inv_32_1 | Chr_05 | Chr_08 | 5.637 | 0.227 | 0.040 | 24.884 | 2.056 | 0.725 | 0.056 | 0.074 | 0.033 |
| L_664 | Inv_21_2 | Inv_32_2 | Chr_05 | Chr_08 | 5.637 | 0.227 | 0.027 | 42.753 | -2.206 | -0.938 | 0.035 | -0.080 | 0.041 |
| L_664 | Inv_22_1 | Inv_30_1 | Chr_05 | Chr_08 | 5.510 | 0.244 | 0.030 | 26.891 | 2.172 | 0.704 | 0.036 | 0.085 | 0.021 |
| L_664 | Inv_22_2 | Inv_54_2 | Chr_05 | Chr_13 | 5.075 | 0.287 | 0.042 | 41.086 | 2.038 | 0.765 | 0.050 | 0.082 | 0.029 |
| L_664 | Inv_24_1 | Inv_30_2 | Chr_06 | Chr_08 | 9.590 | 0.051 | 0.027 | 15.095 | -2.207 | -0.732 | 0.032 | -0.073 | 0.036 |
| L_664 | Inv_24_1 | Inv_32_1 | Chr_06 | Chr_08 | 7.782 | 0.098 | 0.033 | 13.108 | 2.128 | 0.683 | 0.038 | 0.078 | 0.026 |
| L_664 | Inv_24_2 | Inv_40_1 | Chr_06 | Chr_10 | 5.863 | 0.205 | 0.046 | 26.525 | 1.998 | 0.856 | 0.063 | 0.062 | 0.039 |
| L_664 | Inv_24_2 | Inv_43_1 | Chr_06 | Chr_10 | 10.404 | 0.033 | 0.002 | 36.871 | 3.159 | 1.460 | 0.002 | 0.100 | 0.002 |
| L_664 | Inv_24_2 | Inv_43_2 | Chr_06 | Chr_10 | 10.404 | 0.033 | 0.027 | 29.640 | -2.206 | -1.538 | 0.027 | -0.080 | 0.042 |
| L_664 | Inv_24_2 | Inv_54_2 | Chr_06 | Chr_13 | 5.419 | 0.248 | 0.056 | 41.663 | 1.911 | 0.821 | 0.090 | 0.079 | 0.045 |
| L_664 | Inv_24_2 | Inv_9_2 | Chr_06 | Chr_02 | 8.056 | 0.084 | 0.009 | 53.439 | 2.617 | 1.110 | 0.014 | 0.112 | 0.006 |
| L_664 | Inv_29_2 | Inv_48_1 | Chr_08 | Chr_11 | 5.260 | 0.262 | 0.047 | 22.440 | 1.987 | 0.657 | 0.051 | 0.074 | 0.038 |
| L_664 | Inv_29_2 | Inv_56_2 | Chr_08 | Chr_13 | 6.693 | 0.158 | 0.011 | 44.050 | 2.530 | 0.926 | 0.014 | 0.102 | 0.008 |
| L_664 | Inv_30_1 | Inv_43_2 | Chr_08 | Chr_10 | 11.078 | 0.026 | 0.025 | 19.370 | -2.245 | -0.911 | 0.034 | -0.071 | 0.034 |
| L_664 | Inv_30_2 | Inv_54_2 | Chr_08 | Chr_13 | 6.909 | 0.146 | 0.021 | 36.645 | 2.302 | 0.856 | 0.033 | 0.092 | 0.015 |
| L_664 | Inv_32_1 | Inv_43_2 | Chr_08 | Chr_10 | 11.758 | 0.020 | 0.005 | 24.658 | -2.795 | -1.104 | 0.007 | -0.081 | 0.010 |
| L_664 | Inv_32_1 | Inv_45_2 | Chr_08 | Chr_10 | 12.506 | 0.015 | 0.014 | 18.039 | -2.445 | -0.979 | 0.018 | -0.071 | 0.024 |
| L_664 | Inv_32_1 | Inv_48_2 | Chr_08 | Chr_11 | 5.997 | 0.203 | 0.055 | 22.309 | 1.922 | 0.733 | 0.064 | 0.065 | 0.046 |
| L_664 | Inv_32_1 | Inv_6a_1 | Chr_08 | Chr_01 | 10.090 | 0.037 | 0.005 | 18.035 | -2.824 | -0.914 | 0.006 | -0.092 | 0.010 |
| L_664 | Inv_32_2 | Inv_6a_1 | Chr_08 | Chr_01 | 10.090 | 0.037 | 0.022 | 17.799 | 2.296 | 0.831 | 0.023 | 0.082 | 0.015 |
| L_664 | Inv_35_1 | Inv_43_1 | Chr_08 | Chr_10 | 7.463 | 0.114 | 0.052 | 10.963 | 1.946 | 0.625 | 0.056 | 0.071 | 0.040 |
| L_664 | Inv_35_1 | Inv_54_2 | Chr_08 | Chr_13 | 7.496 | 0.118 | 0.014 | 28.018 | -2.458 | -0.924 | 0.016 | -0.072 | 0.023 |
| L_664 | Inv_35_2 | Inv_54_2 | Chr_08 | Chr_13 | 7.496 | 0.118 | 0.014 | 44.673 | 2.456 | 0.949 | 0.020 | 0.104 | 0.009 |
| L_664 | Inv_40_1 | Inv_62_1 | Chr_10 | Chr_14 | 8.615 | 0.072 | 0.013 | 16.848 | 2.473 | 0.796 | 0.017 | 0.093 | 0.009 |
| L_664 | Inv_48_1 | Inv_56_2 | Chr_11 | Chr_13 | 5.037 | 0.289 | 0.049 | 27.668 | 1.966 | 0.734 | 0.066 | 0.068 | 0.040 |
| L_664 | Inv_50_2 | Inv_56_2 | Chr_12 | Chr_13 | 3.966 | 0.419 | 0.058 | 50.063 | 1.899 | 0.737 | 0.094 | 0.079 | 0.044 |
| L_664 | Inv_51_2 | Inv_56_2 | Chr_12 | Chr_13 | 3.966 | 0.414 | 0.058 | 50.063 | 1.899 | 0.737 | 0.094 | 0.079 | 0.043 |
| L_664 | Inv_54_1 | Inv_7_1 | Chr_13 | Chr_02 | 5.762 | 0.215 | 0.044 | 17.385 | 2.016 | 0.646 | 0.055 | 0.075 | 0.034 |
| L_664 | Inv_56_1 | Inv_9_1 | Chr_13 | Chr_02 | 7.200 | 0.119 | 0.018 | 18.118 | 2.365 | 0.764 | 0.025 | 0.088 | 0.013 |
| L_664 | Inv_56_2 | Inv_9_1 | Chr_13 | Chr_02 | 7.200 | 0.119 | 0.049 | 20.630 | -1.966 | -0.734 | 0.066 | -0.063 | 0.063 |
| L_664 | Inv_62_1 | Inv_9_1 | Chr_14 | Chr_02 | 5.760 | 0.223 | 0.046 | 17.689 | -1.994 | -0.638 | 0.056 | -0.067 | 0.060 |
| L_664 | Inv_6a_2 | Inv_7_2 | Chr_01 | Chr_02 | 9.195 | 0.057 | 0.018 | 34.260 | -2.365 | -1.269 | 0.018 | -0.084 | 0.032 |
| L_909 | Inv_21_1 | Inv_35_2 | Chr_05 | Chr_08 | 9.027 | 0.061 | 0.019 | 20.852 | -2.345 | -0.836 | 0.023 | -0.080 | 0.026 |
| L_909 | Inv_21_1 | Inv_54_1 | Chr_05 | Chr_13 | 8.484 | 0.078 | 0.005 | 21.376 | -2.791 | -0.931 | 0.008 | -0.093 | 0.010 |
| L_909 | Inv_21_2 | Inv_35_2 | Chr_05 | Chr_08 | 9.027 | 0.061 | 0.036 | 23.525 | 2.095 | 0.780 | 0.051 | 0.089 | 0.026 |
| L_909 | Inv_21_2 | Inv_37_1 | Chr_05 | Chr_09 | 5.738 | 0.225 | 0.019 | 35.288 | -2.346 | -0.876 | 0.028 | -0.078 | 0.029 |
| L_909 | Inv_25_1 | Inv_44_1 | Chr_06 | Chr_10 | 7.638 | 0.109 | 0.012 | 15.996 | -2.517 | -0.854 | 0.013 | -0.083 | 0.018 |
| L_909 | Inv_25_1 | Inv_45_1 | Chr_06 | Chr_10 | 7.940 | 0.101 | 0.009 | 15.541 | -2.613 | -0.899 | 0.012 | -0.084 | 0.014 |
| L_909 | Inv_25_1 | Inv_46_1 | Chr_06 | Chr_10 | 6.696 | 0.154 | 0.016 | 16.074 | -2.420 | -0.827 | 0.019 | -0.079 | 0.023 |
| L_909 | Inv_25_1 | Inv_47_2 | Chr_06 | Chr_11 | 8.291 | 0.078 | 0.022 | 20.996 | -2.292 | -0.865 | 0.025 | -0.070 | 0.028 |
| L_909 | Inv_25_1 | Inv_48_2 | Chr_06 | Chr_11 | 8.005 | 0.090 | 0.013 | 25.746 | -2.482 | -0.948 | 0.021 | -0.075 | 0.022 |
| L_909 | Inv_25_1 | Inv_49_2 | Chr_06 | Chr_12 | 11.852 | 0.017 | 0.002 | 26.708 | -3.063 | -1.195 | 0.004 | -0.090 | 0.006 |
| L_909 | Inv_25_1 | Inv_53_1 | Chr_06 | Chr_13 | 7.964 | 0.086 | 0.017 | 11.541 | 2.377 | 0.815 | 0.025 | 0.084 | 0.013 |
| L_909 | Inv_25_1 | Inv_53_2 | Chr_06 | Chr_13 | 7.964 | 0.086 | 0.007 | 27.232 | -2.683 | -0.948 | 0.009 | -0.086 | 0.014 |
| L_909 | Inv_25_2 | Inv_47_2 | Chr_06 | Chr_11 | 8.291 | 0.078 | 0.020 | 38.499 | 2.331 | 0.970 | 0.035 | 0.102 | 0.013 |
| L_909 | Inv_25_2 | Inv_48_2 | Chr_06 | Chr_11 | 8.005 | 0.090 | 0.047 | 29.115 | 1.983 | 0.837 | 0.062 | 0.086 | 0.038 |
| L_909 | Inv_2_2 | Inv_62_1 | Chr_01 | Chr_14 | 5.946 | 0.204 | 0.029 | 34.696 | 2.180 | 0.806 | 0.038 | 0.089 | 0.022 |
| L_909 | Inv_2_2 | Inv_62_2 | Chr_01 | Chr_14 | 5.946 | 0.204 | 0.040 | 31.900 | -2.056 | -0.832 | 0.056 | -0.074 | 0.055 |
| L_909 | Inv_31_1 | Inv_40_2 | Chr_08 | Chr_10 | 7.615 | 0.107 | 0.028 | 25.986 | 2.204 | 0.860 | 0.034 | 0.082 | 0.021 |
| L_909 | Inv_31_2 | Inv_40_2 | Chr_08 | Chr_10 | 7.615 | 0.107 | 0.014 | 41.714 | -2.457 | -1.221 | 0.013 | -0.089 | 0.022 |
| L_909 | Inv_32_1 | Inv_40_2 | Chr_08 | Chr_10 | 7.262 | 0.128 | 0.010 | 37.683 | 2.591 | 1.020 | 0.012 | 0.098 | 0.006 |
| L_909 | Inv_32_1 | Inv_44_2 | Chr_08 | Chr_10 | 5.707 | 0.224 | 0.019 | 42.946 | 2.350 | 1.058 | 0.027 | 0.079 | 0.014 |
| L_909 | Inv_32_1 | Inv_45_2 | Chr_08 | Chr_10 | 3.816 | 0.434 | 0.052 | 44.707 | 1.945 | 0.896 | 0.069 | 0.063 | 0.041 |
| L_909 | Inv_32_1 | Inv_62_2 | Chr_08 | Chr_14 | 4.323 | 0.361 | 0.066 | 25.623 | 1.837 | 0.621 | 0.090 | 0.073 | 0.050 |
| L_909 | Inv_32_2 | Inv_40_2 | Chr_08 | Chr_10 | 7.262 | 0.128 | 0.027 | 34.739 | -2.211 | -1.044 | 0.038 | -0.081 | 0.038 |
| L_909 | Inv_35_1 | Inv_45_2 | Chr_08 | Chr_10 | 6.087 | 0.199 | 0.025 | 39.030 | 2.240 | 1.027 | 0.038 | 0.076 | 0.019 |
| L_909 | Inv_35_2 | Inv_40_2 | Chr_08 | Chr_10 | 5.436 | 0.247 | 0.027 | 46.411 | -2.211 | -1.044 | 0.038 | -0.081 | 0.038 |
| L_909 | Inv_35_2 | Inv_53_1 | Chr_08 | Chr_13 | 4.671 | 0.335 | 0.044 | 21.347 | 2.011 | 0.771 | 0.047 | 0.066 | 0.034 |
| L_909 | Inv_35_2 | Inv_53_2 | Chr_08 | Chr_13 | 4.671 | 0.335 | 0.037 | 42.628 | -2.088 | -0.840 | 0.041 | -0.077 | 0.053 |
| L_909 | Inv_40_1 | Inv_47_2 | Chr_10 | Chr_11 | 6.836 | 0.147 | 0.039 | 21.228 | 2.061 | 0.804 | 0.058 | 0.072 | 0.031 |
| L_909 | Inv_40_1 | Inv_48_2 | Chr_10 | Chr_11 | 6.114 | 0.185 | 0.057 | 20.443 | 1.905 | 0.747 | 0.060 | 0.066 | 0.047 |
| L_909 | Inv_44_1 | Inv_49_1 | Chr_10 | Chr_12 | 8.517 | 0.078 | 0.011 | 15.940 | -2.558 | -0.860 | 0.013 | -0.085 | 0.016 |
| L_909 | Inv_44_1 | Inv_49_2 | Chr_10 | Chr_12 | 8.517 | 0.078 | 0.031 | 17.812 | 2.153 | 0.878 | 0.035 | 0.071 | 0.022 |
| L_909 | Inv_44_2 | Inv_49_2 | Chr_10 | Chr_12 | 8.517 | 0.078 | 0.037 | 32.157 | -2.087 | -1.473 | 0.041 | -0.077 | 0.051 |
| L_909 | Inv_4_1 | Inv_44_2 | Chr_01 | Chr_10 | 4.793 | 0.323 | 0.047 | 39.856 | -1.989 | -0.967 | 0.049 | -0.063 | 0.063 |
| L_909 | Inv_49_1 | Inv_45_1 | Chr_12 | Chr_10 | 10.139 | 0.038 | 0.009 | 13.052 | -2.609 | -0.887 | 0.012 | -0.085 | 0.018 |
| L_909 | Inv_49_2 | Inv_45_1 | Chr_12 | Chr_10 | 10.139 | 0.038 | 0.010 | 20.038 | 2.573 | 1.103 | 0.011 | 0.083 | 0.006 |
| L_909 | Inv_45_1 | Inv_9_1 | Chr_10 | Chr_02 | 7.716 | 0.104 | 0.053 | 10.270 | 1.938 | 0.653 | 0.066 | 0.071 | 0.041 |
| L_909 | Inv_49_2 | Inv_45_2 | Chr_12 | Chr_10 | 10.139 | 0.038 | 0.015 | 37.217 | -2.430 | -2.138 | 0.016 | -0.088 | 0.029 |
| L_909 | Inv_49_1 | Inv_46_1 | Chr_12 | Chr_10 | 7.346 | 0.119 | 0.036 | 11.799 | -2.094 | -0.705 | 0.045 | -0.069 | 0.049 |
| L_909 | Inv_49_2 | Inv_46_1 | Chr_12 | Chr_10 | 7.346 | 0.119 | 0.023 | 21.922 | 2.272 | 0.949 | 0.032 | 0.073 | 0.018 |
| L_909 | Inv_49_2 | Inv_46_2 | Chr_12 | Chr_10 | 7.346 | 0.119 | 0.029 | 40.250 | -2.177 | -1.526 | 0.027 | -0.080 | 0.041 |
| L_909 | Inv_49_2 | Inv_47_1 | Chr_12 | Chr_11 | 7.316 | 0.121 | 0.028 | 23.959 | -2.197 | -0.852 | 0.035 | -0.069 | 0.040 |
| L_909 | Inv_49_2 | Inv_47_2 | Chr_12 | Chr_11 | 7.316 | 0.121 | 0.037 | 33.461 | 2.086 | 0.841 | 0.048 | 0.091 | 0.024 |
| L_909 | Inv_49_2 | Inv_48_1 | Chr_12 | Chr_11 | 6.728 | 0.147 | 0.028 | 26.052 | -2.197 | -0.852 | 0.035 | -0.069 | 0.043 |

**1S Fig. Distribution of the *p*-values derived from the omnibus** $\boldsymbol{\chi}^{\boldsymbol{2}}$ **test, the affinity analysis, and the centered Jaccard-Tanimoto-based analysis under the null model.** Histograms showing the density of each bin for the log-transformed *p*-values are complemented with a subplot depicting the cumulative distribution function (CDF).

**2S Fig. Distribution of the *p*-values derived from the omnibus** $\boldsymbol{\chi}^{\boldsymbol{2}}$ **test, the affinity analysis, and the Jaccard-Tanimoto-based analysis under a situation with a deflation of the 9th cell counts.** Those coordinates are based on Fig 1A. There is a shift in the distribution of the *p*-values to the left, given the deviation that this modification implies for the null hypothesis. Histograms showing the density of each bin for the log-transformed *p*-values are complemented with a subplot depicting the cumulative distribution function (CDF).

**3S Fig. Linear Associations Among Various Metrics. This figure illustrates the linear relationships between different pairs of metrics.** Plot A shows the relation between the standardized residuals from the $\chi^{2}$ *post hoc* test and the centered Jaccard / Tanimoto scores. Plot B depicts the relationship between the standardized residuals from the $\chi^{2}$*post hoc* test and the affinity score. Lastly, Plot C demonstrates the linear relationship between the centered Jaccard / Tanimoto score and the affinity score. Each plot includes a trend line, indicating the direction and strength of the linear relationship between the two metrics. The summary of the model is included in the superior edge of each subplot.

**Comparison between LD and co-occurrence metrics in an allele-based analysis**

The analysis of different association metrics revealed distinct patterns in their behavior and frequency dependence. Traditional LD metrics (D and D') and co-occurrence metrics (centered Jaccard-Tanimoto and affinity) showed different properties in terms of stability and frequency relationships (S3 Table).

**S3 Table. Properties of association metrics across different allele frequencies.**

| **Metric** | **sd** | **mean** | **RV** | **EDF** | **r_freq1** | **r_freq2** |
| --- | --- | --- | --- | --- | --- | --- |
| D | 0.03 | 0.07 | 0.46 | 2.52 | -0.00028 | -0.00044 |
| D’ | 0.17 | 0.73 | 0.24 | 2.59 | -0.00019 | -0.00041 |
| cJ/T | 0.07 | 0.14 | 0.53 | 1.31 | -0.25554 | -0.25564 |
| ɑ | 2.02 | 3.09 | 0.65 | 1.00 | -0.00099 | 0.00074 |
| Metrics: **D** and **D’** are routinely used LD metrics in genetic studies. **CJ/T** refers to the centered Jaccard / Tanimoto index. **ɑ** is the affinity score. **sd**: Standard deviation. **RV**: relative variation. **EDF**: Effective degrees of freedom. **r_freq1**: Pearson’s coefficient of correlation of the metric with the allele frequency of the inversion 1. **r_freq2**: Pearson’s coefficient of correlation of the metric with the allele frequency of the inversion 2. | | | | | | |

The traditional LD metrics showed contrasting properties. While D' exhibited the lowest relative variation (0.24) across frequency combinations, both D and D' demonstrated complex relationships with allele frequencies, as evidenced by their high EDF scores (around 2.5). This indicates that while D' may appear more stable, its behavior varies in complex ways with changing allele frequencies.

In contrast, co-occurrence metrics showed higher relative variation but simpler relationships with allele frequencies. The centered Jaccard-Tanimoto index and Affinity had lower EDF scores (1.31 and 1.00 respectively), indicating more straightforward, linear relationships with allele frequencies. The centered Jaccard-Tanimoto index showed moderate negative correlations with allele frequencies (approximately -0.25), while Affinity showed minimal correlation with frequencies.

These results suggest that while co-occurrence metrics may show more variation in absolute terms, their simpler relationship with allele frequencies makes them more interpretable for genotype-level analyses. This is particularly relevant when analyzing genotypic associations, where the biological interpretation of traditional LD metrics may be less direct due to their foundation in haplotype frequencies. Additionally, affinity score showed the most homogeneous surface of values in the frequency space of the pair of inversion analyzed, which is the desired behavior we would expect if the strength of association is fixed (4S Fig).

**4S Fig. Mean value variation of various metrics across the allele frequency landscape defined by two hypothetical inversions.** Uppermost left panel, *D*. Uppermost right panel, *D’*. Bottom left panel, *cJ/T*. Bottom right panel, *α*.
