# Supplementary material for: Are you with me? Co-occurrence tests from community ecology can identify positive and negative epistasis between inversions in Mimulus guttatus
Source: PLoS One. 2025 Apr 28;20(4):e0321253. doi: 10.1371/journal.pone.0321253 (PMC12036897; doi:10.1371/journal.pone.0321253)
Supplement: Attachment — Submitted filename: 2024.12.12_PONE-D-24-52521_review.pdf [file pone.0321253.s008.pdf]

<u>Review:</u> Co-occurrence tests from community ecology can identify positive and negative epistasis between inversions in *Mimulus guttatus*

<u>Summary of work:</u> This manuscript investigates epistasis between inversions in the genome of *Mimulus guttatus*. While genomic structural variants such as inversions have been shown to be relevant to key evolutionary processes like speciation and clinal adaptation, most studies focus on individual inversions in isolation. Here, the authors are interested in whether interactions between inversions result in non-additive effects on fitness, specifically at the level of gametic competition or differential survival/viability.

To do this they champion a novel approach: adapting a framework originally developed in community ecology to analyse species co-occurrence. Given that non-additive fitness effects should manifest themselves as deviations in frequency of inversion co-occurrence relative to expectations derived from individual occurrences, such metrics can be utilized to identify epistasis. Specifically, they use chi-square tests, the Jaccard / Tanimoto index (referred to hereafter as J) and the recently developed affinity score α (Mainali *et al.* 2022) to measure pairwise co-occurrence scores. Having demonstrated that these metrics yield qualitatively similar results, the authors use a machine-learning based network analysis with J to characterize the inversion "interactome" in the species.

<u>Positives:</u> This study addresses a relevant gap in the literature, namely epistatic interactions between inversions. As they have accurately identified, few studies examine non-additive interactions between inversions, and even fewer tackle such a question with such a substantial sample size of inversions (or combinatorial space between them).

Using their novel analytical approach, the authors reliably demonstrate that a few inversion combinations deviate from additivity, though this occurs in only a small portion of combinatorial space (< 10% for all measures tested) for the fitness component of interest. However, these combinations do not interact consistently in the same direction. Some show positive interactions in certain crosses but negative interactions in others, raising questions about whether consistent biological underpinnings exist for this phenomenon.

The authors cleverly avoid the confound of linkage through physical proximity by only considering combinations of inversions on different chromosomes; demonstrate that Mendelian inheritance has no effect on their estimated metrics through null simulations; and take into consideration the potential effects of segregation distortion on measures of epistasis. Lastly, through network analysis, they identify that no higher-order interactions occur beyond the pairwise epistasis observed in this system.

The article is well organized, and the writing is largely clear and coherent, though it could benefit from minor copy-editing. Overall, the study addresses a relevant question and produces meaningful results. However, there are both major and minor concerns that need to be addressed, as outlined below.

Framing: While not a major issue, this point may be relevant to the paper's framing: This manuscript is more a methodological paper about a novel system to measure epistasis that uses inversions as the loci under consideration, rather than one that uncovers something inherent about the biology of inversions. It doesn't really engage with why inversions may be pre-disposed to interact with one another non-additively. Is there something inherent about inversions, perhaps their suppression of recombination, that leads us to predict such epistasis? The closest thing to such theory in the paper is a brief note that if such interactions did exist, they could be particularly relevant in shaping hybrid incompatibilities.

Major Issues:

I identify two sets of major issues in this manuscript.

1. "Why am I with you?" In proposing a novel system to study epistatic interactions, the manuscript does not engage at all with established frameworks, which I see as the most critical issue. Traditionally, epistasis is associated with signatures of linkage disequilibrium. It is possible that the authors have identified why metrics of co-occurrence, such as J and α, are better suited for this context than the coefficient of linkage disequilibrium (D). If so, it is essential that they articulate this argument clearly. Ideally, this should be supported by a comparative analysis of outcomes derived from D-based pairwise and higher-order estimations versus those obtained using J and α. If this has not been considered, and D is in fact a superior (or similar) framework, it should be used in place of J for this analysis. More generally, this would better situate this novel proposal in the context of the work that precedes it.

2. Important ideas / decisions are not described, or where they are – they are not adequately detailed / justified. I note some important cases of this here:

    a. J, α indices: Although central to the thesis, the authors do not explain how either of these statistics work. As a manuscript proposing a new system of analysis, it is necessary that the authors provide at least a rudimentary explanation, preferably with a toy example to guide the reader's intuition.
    b. Identifying a pattern of selection associated with segregation distortion (SD): The authors briefly mention using a "sequential χ^2 analysis" to detect selection patterns but fail to describe how this method determines the underlying form of selection. Further, it would be useful to describe why we expect SD to influence co-occurrence. This is briefly touched upon much later in the discussion.
    c. Multi-way interactions and how they may be shown using network analysis: The authors propose network analysis as a tool to identify higher-order interactions between inversions. However, they do not explain how network analysis achieves this. A simple illustrative example, such as what results would look like under a hypothetical 3-way interaction, would greatly aid reader comprehension.

d. Binarized analyses:
    i. SD and its downstream effects: When assessing the effects of SD on epistatic interactions, the authors use a binary variable (significant/non-significant SD) as the predictor and count of significant/non-significant interactions (along with mean p-values across combinations) as the independent variable. This choice is not explained – an easy alternative could have been to analyse raw *J* scores using raw SD values as a predictor. If a relationship did exist, residuals extracted from this model could have provided a measure of co-occurrence while controlling for SD. It is possible that I am overlooking a statistical obstacle in proposing this – but such an issue would be worth explaining.
    ii. Metrics congruence: The authors use a similar approach to demonstrate congruence between the metrics; however, this congruence could just as easily be assessed using the raw metric scores. Although thematically linked to the rest of this major issue, this point is not a major concern. Here, there is some justification provided; this allows to test if significant hits between the various metrics are robust – but general congruence between these systems would also support the selection of a single system to base the remaining results upon. Additionally, they do compare residuals from these estimates using linear regressions.
e. Bootstrapped analyses: For several statistical tests, the authors bootstrap-sample their data to conduct their analysis. While there is nothing inherently wrong with this approach, the manuscript would benefit from a clear rationale for its use. For example, when estimating co-occurrence metrics and their p-values, it seems feasible to perform the calculations directly on the full dataset without resampling. Additionally, while bootstrapping should yield a distribution of p-values, the authors present their significant/non-significant binarization (for testing metric congruence and relationship with SD) as if they are working with a single p-value. This approach is not described, and the process for summarizing or selecting p-values requires clarification.
f. Simulated p-value distributions: The authors conduct simulations to account for potential complications arising from Mendelian inheritance. In addition to null simulations, they simulate scenarios with epistatic interactions to illustrate the expected outcomes for their metrics. However, they select rather extreme selection coefficients without providing a justification for this choice. It appears these values were chosen to ensure that deviations from additivity would be detectable by their metrics, thereby highlighting the relevant submatrices being affected. If this was indeed the rationale, it should be explicitly stated to clarify the reasoning behind their approach.
g. p-value corrections: The authors report their method of p-value correction to account for multiple testing, but in the very next sentence explain that they discard these corrections as they are too stringent. This suggests that the remainder of their analysis is likely vulnerable to type-1 errors.

<u>*Minor Issues:*</u>

1. The authors identify that the genetic family / cross identity is an explanatory variable in determining epistasis and the direction thereof. One possible route to such a result could be poor randomization – for instance, if the offspring of each cross was treated together / maintained in the same location of a greenhouse etc. In this context, it would be valuable to note down the systems of randomization used to ensure that this was not the cause for the effects observed. More generally, it would be useful to for the authors to speculate – why is the genetic family so critical here?
2. Figure 3 is too pixelated to follow – even digitally. Figure labels are mismatched with the text, see lines 144, 149 for instance. Figure captions repeatedly mention the source of the populations used – but this isn't necessary. Additionally, overall figure titles for supplemental figures would be helpful to distinguish plots from various simulations.
3. Line 123: *"Each inversion is split in two entities, homozygous and heterozygous states"* – the phrasing here is unclear. Doesn't this simply mean that each locus is coded as *Std*, *Inv*, or *Het*?
4. Line 133: "*We also categorized the pattern of selection in case of SG**... *proposed by Fu et al. (2020).*" – The term *SG* appears to be a typo and should read *SD*.
5. Line 134: *"The segregation distortion value (SDV), defined as the negative decimal logarithm of the SD-associated values."* – The phrasing here is incomplete or awkward. Perhaps rephrase as: "The SDV is defined as the negative decimal logarithm of the SD-associated values.*"* Alternatively, if the sentence is missing a clause, this should be clarified to ensure the definition is complete and comprehensible.
6. Line 149: *"For the former method"* – the phrasing here is unclear, as the "former method" was already described as using a different package. Explicitly naming the method would make this section easier to follow and eliminate ambiguity.
7. Lines 343-358: "*Distinct patterns in frequency of inversion combinations observed*": The authors enumerate frequencies of homozygous-homozygous, homozygous-heterozygous, and heterozygous-heterozygous combinations. Firstly, it would be more informative to express these frequencies as a percentage of the total number of possible valid combinations within the sample. Secondly, the authors ascribe positive or negative tendencies of associations based on very equivocal data—for example, describing 9 negative interactions versus 7 positives as a tendency toward negative interactions seems tenuous. Lastly, the manuscript lacks predictions or post-hoc explanations regarding the biological relevance of these patterns. For instance, is there a specific reason or biological justification for heterozygous-homozygous combinations being more frequent than the others? In fact, is there a reason to believe that these outcomes are not simply stochastic?
8. Line 465: "*it could shred* light*" should be shed.
9. Line 505: "*Something that results evident from a quick inspection*" could be "something evident"
10. Line 519: "*it can be seen a significant effect for the heterogeneity test*" could be "can be seen as a".
11. Line 530: "*are regions ... parts of the genome.*" could be "*are regions ... parts of the genome?*"

12. Lines 539-544: Can be avoided altogether.
    a. "*we calculated pairwise statistics between pairs of inversions on the same chromosome indicated a strong attraction or positive epistasis*" could be "pairwise statistics calculated between …. epistasis". Additionally, this is rather loose usage of the term epistasis. These clearly co-occur due to physical linkage.
    b. Likewise "*pairwise contrasts between different states of dosage (heterozygous against homozygous) turned out to be strongly repulsive (negative epistasis) … given by the fact that a given plant cannot be heterozygous and homozygous for the same inversion simultaneously*" is also trivial and does not need to be reported.
