# Supplementary material for: Are you with me? Co-occurrence tests from community ecology can identify positive and negative epistasis between inversions in Mimulus guttatus
Source: PLoS One. 2025 Apr 28;20(4):e0321253. doi: 10.1371/journal.pone.0321253 (PMC12036897; doi:10.1371/journal.pone.0321253)
Supplement: Attachment — Submitted filename: 2025.02.04_PONE-D-24-52521_review_round2.pdf [file pone.0321253.s010.pdf]

Response to edited MS*:** **Co-occurrence tests from community ecology can identify positive and negative epistasis between inversions in *Mimulus guttatus

I am satisfied that meaningful improvements have been made through revisions in line with the AE's guidance. While their engagement with LD remains somewhat underdeveloped / could be better articulated, it is an improvement over their previous version. A more direct comparison using LD as a metric—supported by statistical phasing of haplotypes—would have resulted in much stronger understanding. Although not an obstacle to scientific understanding, the language in the manuscript still requires substantial proofreading. I recommend accepting, given some minor revisions, for eg. acknowledging certain caveats. In addition, the extended methods section of the supplement appear to be missing, along with the details of how they carried out the simulation based comparison, and should be included prior to acceptance.

Detailed comments inline (authors' responses to original review comments in italics).

Major issue #1: Engagement with LD, comparative analysis.

Our choice of co-occurrence metrics over LD metrics was motivated by both biological and statistical considerations. First, our analysis focuses on genotypic associations rather than haplotypic relationships, making co-occurrence metrics more directly applicable to our research question. (Expanded within text[1,2])

The rationale to prioritize genotypic associations could be better established here. It is possible that there are certain relationships that exist exclusively between certain genotypic states due to non-additivity of allelic state information, but is there good reason to argue that this is common? That said, the authors do describe a rationale for their choice, and it seems sufficiently reasonable to be opened to broader debate.

Second, while our analysis shows that LD metrics can be stable (relative variation of D' = 0.238), they exhibit more complex relationships with allele frequencies (EDF ≈ 2.5) compared to co-occurrence_metrics (EDF ≈ 1.0-1.3). This simpler frequency relationship makes co-occurrence metrics more interpretable for our genotype-level analysis.

Nevertheless, we described the performance of those metrics (see updated Supplementing Information) based on simulated scenarios using as criteria the stability of the metrics under a fixed level of association, in terms of complexity, stability, and relative variation. We obtained mixed results, briefly stated earlier, and we describe them as supplementary materials.

As far as I can see, neither simulation methods/parameters nor the calculations used to make subsequent measurements are described in the supplement. I am unable to follow what has been reported.

It is worth noticing that we could not employ directly the LD metrics on the same way we conducted our analysis, because we were searching for associations between inversions in terms of genotypes and LD metrics work over allele frequencies. For the simulations, we defined

hypothetical inversions and applied the co-occurrence metric in an allele focused manner, to make the results comparable.

In addition to the stated response, the authors add to this in text[2], describing the need for phased data to know haplotype of origin. It would be valuable to note as a caveat here that statistical phasing of genomes has become both increasingly common and accurate.

<u>Major issue #2:</u> Insufficient explanations

2a. J, α indices: *We included an explanation of the metrics as part of the Extended Methods of the paper. We also included a toy figure to show the idea behind those metrics.*

The authors add a description in the introduction[2], and a toy example as promised through fig.2. However, the status of the 'Extended Methods' section is unclear. I am not able to locate such a section in the “supporting information” file. Perhaps this was intended as a supplementary section but omitted accidentally.

2b. SD and co-occurrence: *A description of the sequential Chi-Squared analysis is further developed in the Extended Methods. Also, a more immediate explanation about why we were expecting to see a relation between SDV, and the co-occurrence pattern was included was included in materials and methods.*

As noted above, the extended methods were not available for review. Within the methods section, the only additional explanation is rather vague - a reference to complex relationships between genes[3]. As in the original MS, the rationale for this is explained more clearly in the discussion, though it would be more effective if presented earlier in the manuscript.

2c. Network analysis: *Analysis included now in the paper to detect third and fourth order motifs through the networks.*

Done as promised[5].

2d. Binarized analysis: *The binarized step can be omitted, given that the strong relation evinced in the regression analysis is enough and nuanced to both demonstrate the congruence between methods and the justification of moving forward with only one of them in the visualization and network analysis steps.*

Done as promised.

2e. Bootstrapped analysis: *Clarifications were provide and the binarized analyzed was also removed, given that is redundant with the results directly derived from the regression analysis between metrics.*

Done as promised[6].

2f. Simulated p-value distributions: *We clarified the rationale under the selection of such coefficients. "These specific survival rates (0.2 for deflation, and 1.0/0.7 for inflation/deflation) were deliberately chosen to be pronounced in order to clearly demonstrate the ability of our statistical frameworks to detect deviations from additivity. While such extreme values may be less common in natural populations, they serve an important methodological purpose in validating our analytical approach by producing clear signals that highlight which submatrices are affected under different scenarios of non-additive interactions. This allows us to establish proof-of-principle for our methods before applying them to real data, where the deviations may be more subtle."*

Done as promised.

2g. Type I errors: *Type I errors are indeed a vulnerability of the analysis. We tried several approaches in this regard, but even the least stringent corrections and FDR control were unable to accommodate the complex structure and dependency within our data. Future efforts must be made in order to design a more appropriate correction method, which should take in consideration the boundaries of the p-values obtained through permutational or bootstrap approaches, the non-independency between tests derived from the same 3x3 contingency table, and most importantly, the non-uniformity of the p-value distribution after the midpoint 0.5, which is essential in the estimation of p0 in FDR methods like the q-value correction. Nevertheless, applied as exploratory analysis to detect associations, these pipelines offer a way of selecting targets for further inspection and statistical testing.*

Done as promised, and explicitly noted in the methods as a caveat[4]. The rest of explanation included in this response to the reviewers would be valuable to include in the section on future research directions.

Minor issues:

Omissions have been made as per suggestions. I don't engage here with the minor issues about language errors – but the manuscript still requires proofreading.

[1] Lines 196-205:

Furthermore, a significant distinction from traditional linkage disequilibrium (LD) measures is that these two indexes enable us to analyze the relationship between inversions at the genotypic level (Fig 2). In contrast, traditional methods, such as D and D', focus on a broader allele scope through haplotypes. This genotypic scope is preferred when using Chi-squared approaches and odd ratios test to avoid potential biases introduced by summing allele frequencies [29]. Nevertheless, the main reason we avoided the direct use of LD metrics is related to their variability with the allele frequencies at each locus and linear to non-linear shifts in the LD structure between loci [8]. We included a simulation-based contrast between two widely used LD metrics and the co-occurrence indexes employed in this study, which is explained in the Extended Methods and presented as Supporting Information.

[2] Lines 61-70:

The fact that D measures co-occurrence at the allelic level is noteworthy because genotype data is routinely collected on diploid individuals where the phase alleles at different loci (whether they reside on the maternal or paternal haplotype) is not known. We here consider the centered Jaccard-Tanimoto index (cJ/T) [11,12] and the affinity score [13] because they measure associations at the scale of the diploid genotypes. The cJ/T quantifies association by comparing observed co-occurrence against a null expectation of independence, distinguishing positive ($cJ/T > 0$) and negative ($cJ/T < 0$) associations. Similarly, the affinity score, expressed as a log odds ratio (α), captures co-occurrence tendencies, with positive values indicating coupling and negative values suggesting genetic repulsion. These methods provide a nuanced framework for understanding complex genetic interactions, particularly in scenarios where traditional LD measures may be insufficient.

[3] Lines 142-147:

We used the segregation distortion value (SDV), defined as the negative decimal logarithm of the SD-associated p values, to explore the relation between the individual tests and the pairwise contrasts performed later. We evaluated this association because SF involves complex relationships between genes, which can shape the transmission advantage of certain haplotypes (determined in our context by the combination of inversions) and affecting their genetic associations.

[4] Lines 191-195:

Nevertheless, it is important to notice that these measures also are susceptible to false discoveries common in genomic applications, and careful care must be taken in the interpretation of the results where independence between tests is not achieved in all cases (e.g. there will be four tests associated to the same pair of inversions, as seen in Fig 1 after decomposing the 3x3 table).

[5] Lines 153-157:

Next, we extracted from the networks the subgraphs defined by edges with a raw index-associated p-value less than 0.05. With those subgraphs, we assessed significant motifs of third and fourth order (e.g., triangles, cycles, etc.). In this context, a motif represents an association pattern defined by nodes connected in a specific manner with a frequency higher than expected by chance. The significance of the nodes can be assessed using a permutational procedure rewiring each network several times and counting the number of times that each motif appears. That way, the null distribution of counts per motif can be derived, and with it, the probability of getting a count as extreme or more extreme than the observed one for each motif under the null hypothesis (randomness). For simplicity, we evaluated overrepresented motifs, setting a threshold of a minimum count of 200. Scarce motifs were left out. Having null distributions, it is straightforward to determine the most significant motifs across networks.

[6] Lines 153-157:

We used chisq.test from the base R package and computed the p-value associated to the $\chi 2$ statistic through bootstrapping (10 000 iterations). We did not use the asymptotic approach given that for some tests, we observed small expected frequencies, which leads to a poor approximation of the $\chi 2$ statistic to its theoretical distribution.
