# Supplementary material for: Are you with me? Co-occurrence tests from community ecology can identify positive and negative epistasis between inversions in Mimulus guttatus
Source: PLoS One. 2025 Apr 28;20(4):e0321253. doi: 10.1371/journal.pone.0321253 (PMC12036897; doi:10.1371/journal.pone.0321253)
Supplement: Attachment — Submitted filename: Response to reviewers.docx [file pone.0321253.s011.docx]

**Journal requirements**

1. When submitting your revision, we need you to address these additional requirements.

Please ensure that your manuscript meets PLOS ONE's style requirements, including those for file naming. The PLOS ONE style templates can be found at

https://journals.plos.org/plosone/s/file?id=wjVg/PLOSOne_formatting_sample_main_body.pdf

and

https://journals.plos.org/plosone/s/file?id=ba62/PLOSOne_formatting_sample_title_authors_affiliations.pdf

**A/:** We followed the guidelines, and the manuscript now should meet all the requirements.

2. We note that the grant information you provided in the ‘Funding Information’ and ‘Financial Disclosure’ sections do not match.

**A/:** We fixed the problem. Right grant is now included: NSF grant MCB-1940785

When you resubmit, please ensure that you provide the correct grant numbers for the awards you received for your study in the ‘Funding Information’ section.

**A/:** Fixed.

3. Thank you for stating the following financial disclosure:

National Institute of General Medical Sciences (NIGMS/NIH) under award number P30GM145499.

Please state what role the funders took in the study. If the funders had no role, please state: "The funders had no role in study design, data collection and analysis, decision to publish, or preparation of the manuscript."

If this statement is not correct you must amend it as needed.

Please include this amended Role of Funder statement in your cover letter; we will change the online submission form on your behalf.

**A/:** That financial statement was included by error. We are sorry.

4. Please note that your Data Availability Statement is currently missing the repository name. If your manuscript is accepted for publication, you will be asked to provide these details on a very short timeline. We therefore suggest that you provide this information now, though we will not hold up the peer review process if you are unable.

**A/:** Data Availability Statement added in the manuscript.

5. When completing the data availability statement of the submission form, you indicated that you will make your data available on acceptance. We strongly recommend all authors decide on a data sharing plan before acceptance, as the process can be lengthy and hold up publication timelines. Please note that, though access restrictions are acceptable now, your entire data will need to be made freely accessible if your manuscript is accepted for publication. This policy applies to all data except where public deposition would breach compliance with the protocol approved by your research ethics board. If you are unable to adhere to our open data policy, please kindly revise your statement to explain your reasoning and we will seek the editor's input on an exemption. Please be assured that, once you have provided your new statement, the assessment of your exemption will not hold up the peer review process.

**A/:** Data is fully accessible without any restriction. It is part of the public repository where the code is stored.

6. Please amend either the abstract on the online submission form (via Edit Submission) or the abstract in the manuscript so that they are identical.

**A/:**

7. Please upload a new copy of Figure 3 as the detail is not clear. Please follow the link for more information: https://blogs.plos.org/plos/2019/06/looking-good-tips-for-creating-your-plos-figures-graphics/" https://blogs.plos.org/plos/2019/06/looking-good-tips-for-creating-your-plos-figures-graphics/

**A/:** Figure was removed given that its information is covered with the linear models adjusted in the congruence between methods section. As suggested by one of the reviewers, this binarized analysis was omitted.

**Editor Comments**

**Main points**

1. Provide better explanation for the metrics, as they are novel to the PopGen community. Can be done with extended methods, or supplemental methods. In the intro, the network is presented as an alternative approach to the cooccurrence stats, but here it is used as a follow up analyses/visualization.

**A/:** This was an excellent suggestion. We included a supplemental methods file to cover the theoretical basis of the co-occurrence metrics employed. Also, the use of the network approach was clarified and improved.

2. Elaborate on strengths/weaknesses of these methods to detect coupling vs repulsion (suggest using the LD metric vocabulary).

**A/:** A brief discussion of the advantages and potential flaws of the indexes were included in the Materials and Methods section of the paper. Thank you so much for the suggestion.

3. Can you compare the performance of the metrics with established estimators, like linkage disequilibrium (D and D’). We are not ask for full scale benchmark analyses, but comparing the outputs of the different metrics can illustrate the nature of the signals they are best equipped to identify. Mention in discussion that benchmark analyses on simulated data with various properties (missingness, patterns of coupling, repulsion, recombination rates etc.) would be an ideal next step.

**A/:** This is a good point. We included a simulation-based contrast to illustrate the performance of these two metrics in relation to the LD framework. Nevertheless, it should be noted that LD metrics are best suited for haplotype-based analyses, where the data is analyzed at allelic level. In our case, we were analyzing the inversions at genotypic level. To perform a meaningful contrast, the co-occurrence metrics were applied at the allele level assuming HWE. It will be released as a complementary analysis to improve the integration with LD metrics.

As a summary, we can state that: our choice of co-occurrence metrics over LD metrics was motivated by both biological and statistical considerations. First, our analysis focuses on genotypic associations rather than haplotypic relationships, making co-occurrence metrics more directly applicable to our research question. Second, while our analysis shows that LD metrics can be stable (relative variation of D' = 0.238), they exhibit more complex relationships with allele frequencies (EDF ≈ 2.5) compared to co-occurrence metrics (EDF ≈ 1.0-1.3). This simpler frequency relationship makes co-occurrence metrics more interpretable for our genotype-level analysis.

4. Can you synchronize the terminology and labelling of the different methods? Like between Figure 1 and 2, and text for the simulation methods, it is a bit hard to follow which method is which. Perhaps it is just an issue of improving Figure 2 and its legend ? (don’t need to state “P-values“ in heading of all 9 panels).

**A/:** Yes, that is a good step forward. It is true that it is hard to follow the text without more clarification. The ‘p-value’ was removed from all the panels. Also, the organization was improved by adding a color-based system to identify the omnibus Chi-square test, the affinity-based results, and the Jaccard-based results. Same modifications were made to the supplementary figures.

5. Provide a well annotated R script (GitHub ideally) that explains the steps in the analyses, with well described (metadata) dataset. This will increase likelihood of the methods being picked up by other groups.

**A/:** The annotation of the main and auxiliary R scripts was improved. Thank you so much for the recommendation. They are all available in the GitHub repository released alongside the paper.

6. If the co-occurrence patterns depend strongly on Cross, then you are reporting higher order interactions. Cite literature on those (rather than organism specific papers), like <https://www.sciencedirect.com/science/article/pii/S0168952513000218?via%3Dihub>

**A/:** The paper you recommended was extremely useful for strengthening the discussion of our results (cite added) related to the context dependent nature of the interactome. Thank you so much.

**Intermediate points**

1. Think it may be helpful to generate a figure for the data on 336-242, to demonstrate how the numbers look for the 3x3 tables. This may provide a better link to the graphics following! Your call!

**A/:** Yes, it will be a great addition to a representation of the data. It is included now in Fig 1.

2. Check wording and grammar of the entire manuscript, see our minor points!

**A/:** Checking of grammar and wording done.

**Minor points**

1. Line 45: Don’t agree with the last part of sentence “In genetics, non-random relationships between loci, described as linkage disequilibria, are measures of co-occurrence”. The non random relationships are not measures!

**A/:** We agree. Wording corrected.

2. Line 49: Can you reword “Attraction or repulsion of species in their location.”

**A/:** We reworded the sentence.

3. Line 58: Metrics?? “The co-occurrence techniques.”

**A/:** Most definitely ‘metrics’ is the word to use here. Thank you.

4. Line 64: Drop “of” “In our study species of *Mimulus guttatus*.”

**A/:** We did so.

5. Line 77: Provide references ”Network analysis is an area of active development in biology.”

**A/:** Two references were added to support the statement.

6. Line 79: Singular or plural idea(s)? “The underlaying basic ideas.”

**A/:** It should be in singular. It was fixed.

7. Line 104: Which 10 genomes? “The ten genomes were derived.”

**A/:** Statement clarified. Those genomes were employed to detect the inversions employed in this paper.

8. Line 130: “if genotype frequencies” these are all inversion genotypes?

**A/:** Statement clarified. Now it reads: “ To test for segregation distortion (SD) at individual inversion loci (Fig 1A), we performed a goodness-of-fit test to determine if inversion genotype frequencies (standard homozygous, heterozygous, and inverted homozygous) followed expected Mendelian proportions for an F2 population (0.25/0.50/0.25).”

9. Line 205: How many datapoints were in this comparison dataset? “also detect signals derived from linkage.”

**A/:** The number of data points in this case is simply the number of valid tests we performed in the 2x2 framework. A note in the figure legend was added to clarify this point. In relation with the linkage (in the sense of physical linkage), if the results for inversions in the same chromosome are explored, it can be seen how these tend to co-occur more frequently than by chance. Those inversions have elevated and positive scores for affinity (value 10 or very close to 10, being 10 the capped maximum in the log ratios test) and centered Jaccard / Tanimoto. We do not include those results in the paper because we omit them, because physical linkage alone can explain these interactions (we only care for interchromosomal interactions)

10. Line 211: Used rather than adjusted? “we adjusted robust linear regression instead.”

**A/:** Wording fixed following your recommendation.

11. Line 247-251: Reword this section, introduce the permutation approach earlier in the description.

**A/:** Suggestion implemented following your suggestion. Permutation approach moved before some other support statements related to the decision we made.

12. Line 258: “in six other crosses where it displays no distortion.” Or state directly, “where both types of homozygotes are seen”?

**A/:** Statement re-worded.

13. Line 274: Table 1. Can you annotate the p values in different way? X21 followed by column with its p values, and then X22? And a column that has all the same values is useless, i.e. “Selection”

**A/:** Modifications made following your suggestions.

14. Line 276: Maybe reword title to “Testing of methods via simulation(s??)”?

**A/:** Recommendation of title adopted. Thank you so much.

15. Line 286: Maybe say “counts” were “deflated”, not cell?

**A/:** Fixed.

16. Line 309: “Of the valid contrasts…” sentence is convoluted. Can this be subdivided into 2 or more sentences or reworded?

**A/:** Fixed. Now it reads: “Among contrasts that distinguish between homozygous and heterozygous inversion states, we detected 355 significant associations (~9% of valid combinations) using at least one of three analytical approaches: Jaccard similarity, affinity coefficients, or post-hoc tests (based on uncorrected p-values).”

17. Line 318: Check reference to supplement II.

**A/:** Checked!

18. Line 320: Any cases of the opposite? “but the” instead of “and the”?

**A/:** Suggestion implemented. In relation with the opposite scenario, it can be seen in the Fig 4, for those edges that are significant for the Chi_squared test but not for the Jaccard index.

19. Line 366: Was there good or poor congruence? Consider changing the section heading “Congruence between methods” or first sentence “The congruence between the 2x2 table-based methods is depicted in Fig. 3 and Fig. 3S.” to better explain the main result of this section.

**A/:** There was a good congruence between methods (now added to the results). The section was re-worded following your suggestions. Thank you so much. Notice that congruence now is assessed using the linear regression results, following suggestions of one of the reviewers (binarized analysis are not necessary to show the point).

20. Line 371: “robust”, is this word needed? Anything specifically robust about the regression?

**A/:** Yes, in this context, it refers to a regression analysis designed to be less sensitive to outliers and violations of statistical assumptions. It was necessary to implement it given that the residuals of the ordinary regression implemented first were not normally distributed.

21. Line 367 and Figure 3: It is unclear what is shown in the figure (legend missing details, what is above and below diagonal. Labels on figure to small to read, question if inset color panel is needed for all 9? Make start of Figure 3 legend more transparent “good correspondence was between…”

**A/:** Figure is no longer part of the results. It message can be conveyed directly with the regression results in Supporting Information.

22. Line 384: Isn’t this more of a visualization of the results from the previous section?? Heading “Visualization of co-occurrence as inversion networks” or some version of this?

**A/:** True. Formal network analysis included! Following reviewer suggestions, now we analyzed the motifs in the networks to search for significant patterns (third and fourth order motif included).

23. Line 390: Question of wording ”there were significant patterns”, maybe “signal”?

**A/:** Phrase re-worded.

24. Line 391: “Strongest signals were in lines 155”

**A/:** Suggestion implemented.

25. Line 439: “individuals” rather than “organisms”

**A/:** Change made following suggestion.

26. Line 442: Wording, “Also, inversions can be central elements in our understanding about”, maybe drop the “our understanding”?

**A/:** “our understanding” was dropped.

27. Line 453: “interactome” is not the right word here, find another. Also fix in other places in MS.

**A/:** We agree. Interactome has been adopted in a molecular sense for protein-protein interactions and so forth. We replaced the word all along the manuscript.

28. Line 456: “induced” another verb may be better!

**A/:** Change made following your suggestion.

29. Line 470: Reword “although often not across all crosses” was it not so that this was mainly in few crosses?

**A/:** Change made following your suggestions.

30. Line 479: Check sentence structure and placement of reference.

**A/:** Fixed.

31. Line 482: Strange wording “…where the higher count of plants was found in the homozygous”. Can you rephrase. “more plants were homozygous for inversion 49 …”?

**A/:** Fixed following your suggestion.

32. Line 485: “effective population size”?

**A/:** Yes. Fixed following your suggestion.

33. Line 491: Wording? “Applied to in this study”

**A/:** Wording fixed.

34. Line 507: Add citations to papers about higher order epistasis, see above.

**A/:** The citations were added. Thank you very much.

35. Line 511: “We investigated one such example in detail” rather than “Figure 5 provides a particular example of variability of interactions.” Or some other more descriptive version of this.

**A/:** Wording fixed.

36. Line 518: Reword, in particular clunky text at “In the case of the inversion 32 in homozygous state and the inversion 40 in heterozygous state, it can be seen a significant effect for the heterogeneity”

**A/:** Statements re-worded.

37. Line 528: “strong” not “string”?

**A/:** Fixed.

38. Line 530: Verb missing…”are?”

**A/:** Fixed.

39. Line 533: Maybe we just need more power, or that the decencies of SDV and inversion interactions are diverse, on case by case basis?

**A/:** We think that is more associated with the ideas stated in Wagner *et al*. (2007), related to the modular nature of genetic architectures. Nevertheless, it could also be related with the cross-specific interactions we observed.

40. Line 565 and 570: Add references to these sections, so to bring attention to these methods through scholar working on these topics.

**A/:** Additional references added.

41. Line 579: Or how inversion combinations are depleted or enriched in hybrids between species or subspecies.

**A/:** That is another possible application in this context. Thank you.

42. Line 584: Can also be used to study combinations of specific alleles of the genes within inversions, both within one inversion or across inversions.

**A/:** Yes, that is embedded in the current analysis, given that the inversion lock such combination of alleles.

43. Line 628: Wording ”in special to” ?

**A/:** Fixed.

**Reviewers**

Reviewer 1:

1. Co-occurrence analyses are usually used to quantify species loss and gain across communities, to investigate patterns of antibiotic cross-resistance, and to identify mechanistic similarities among diseases. Authors need to elaborate the use of co-occurrence analysis for attraction and repulsion between different inversions. This is particularly important as the focal theme of the study is to analyze the epistatic interaction between the inversions. Authors may add a note on the perceived advantage of using Jaccard-Tanimoto index over Sørensen-Dice, and Simpson methods.

**A/:** Yes, we see your observation as more than accurate. More context was added in this regard. Also, a note about the perceived advantage of the metrics employed was included now in the Materials and Methods section.

2. For Table I, authors have used P-values as suggested by the analysis software, which means raw p-values have been utilized. This is not appropriate, kindly improve the presentation and add scientific P-values < 0.05." This is crucial as the co-occurrence analysis focuses only on the significant positive associations.

**A/:** For this analysis, the magnitud of the *p*-value (how much lower than 0.05) is critical. We kindly ask to leave the table with the p-values in the current notation.

3. For the network analysis, some other libraries and approaches can be used to achieve the comprehended results of the Jaccard indexes and taken as a reference for building interaction. You can find alternatives and consider NetworkX in Python instead of igraph in R.

**A/:** It is true that other alternatives are also available to achieve the same results we got using igraph in R. Nevertheless, we prefer to preserve the current approach, given that the entire pipeline could be replicated by using a single command (Rscript main.R) and language (R in this case). We could introduce an alternative pipeline to take the results derived from the R scripts into python, but it seems an extra complication considering the good performance of igraph (which is a powerful and widely used library for network analysis and visualization within the R environment).

4. The techniques or the extensions authors have used vis a vis R language is not described in the method section. Authors need to emphasize and add a description regarding the usage of R or R Studio for the larger benefit of the readers.

**A/:** A statement has been added related to the version of R and RStudio employed. Also, a description of the co-occurrence metrics employed in the study has been added as Extended Methods.

5. Please indicate the type of inversions used for the analysis (paracentric / pericentric). Additionally, a note on the adaptive role or potential of the same needs to be added to the discussion section.

**A/:** In the case of our inversions, we only have paracentric inversion. Even in the case of the biggest ones (one inversion in linkage group 6 and the other in linkage group 11) do not include the centromere.

6. Authors may consider the following references to incorporate in the revised manuscript:

(I) Chromosome inversions and linkage disequilibrium in Drosophila. Current Science 94:459-64.

(ii) Hundred years of research on inversion polymorphism in Drosophila. Current Science 117:761-775.

**A/:** Those are excellent papers. We included both in the main text as references.

Reviewer 2:

**Major issues**

1. “Why am I with you?” In proposing a novel system to study epistatic interactions, the manuscript does not engage at all with established frameworks, which I see as the most critical issue. Traditionally, epistasis is associated with signatures of linkage disequilibrium. It is possible that the authors have identified why metrics of cooccurrence, such as *J* and *α*, are better suited for this context than the coefficient of linkage disequilibrium (*D*). If so, it is essential that they articulate this argument clearly. Ideally, this should be supported by a comparative analysis of outcomes derived from *D*-based pairwise and higher-order estimations versus those obtained using *J* and *α*. If this has not been considered, and *D* is in fact a superior (or similar) framework, it should be used in place of *J* for this analysis. More generally, this would better situate this novel proposal in the context of the work that precedes it.

**A/:** This is an excellent point. Our choice of co-occurrence metrics over LD metrics was motivated by both biological and statistical considerations. First, our analysis focuses on genotypic associations rather than haplotypic relationships, making co-occurrence metrics more directly applicable to our research question. Second, while our analysis shows that LD metrics can be stable (relative variation of D' = 0.238), they exhibit more complex relationships with allele frequencies (EDF ≈ 2.5) compared to co-occurrence metrics (EDF ≈ 1.0-1.3). This simpler frequency relationship makes co-occurrence metrics more interpretable for our genotype-level analysis.

Nevertheless, we described the performance of those metrics (see updated Supplementing Information) based on simulated scenarios using as criteria the stability of the metrics under a fixed level of association, in terms of complexity, stability, and relative variation. We obtained mixed results, briefly stated earlier, and we describe them as supplementary materials. It is worth noticing that we could not employ directly the LD metrics on the same way we conducted our analysis, because we were searching for associations between inversions in terms of genotypes and LD metrics work over allele frequencies. For the simulations, we defined hypothetical inversions and applied the co-occurrence metric in an allele focused manner, to make the results comparable.

2. Important ideas / decisions are not described, or where they are – they are not adequately detailed / justified. I note some important cases of this here:
a. J, α indices: Although central to the thesis, the authors do not explain how either of these statistics work. As a manuscript proposing a new system of analysis, it is necessary that the authors provide at least a rudimentary explanation, preferably with a toy example to guide the reader’s intuition.

**A/:** Excellent point raised. We included an explanation of the metrics as part of the Extended Methods of the paper. We also included a toy figure to show the idea behind those metrics.

b. Identifying a pattern of selection associated with segregation distortion (SD): The authors briefly mention using a "sequential 𝜒² analysis" to detect selection patterns but fail to describe how this method determines the underlying form of selection. Further, it would be useful to describe why we expect SD to influence co-occurrence. This is briefly touched upon much later in the discussion.

**A/:** This is a very welcome suggestion. A description of the sequential Chi-Squared analysis is further developed in the Extended Methods. Also, a more immediate explanation about why we were expecting to see a relation between SDV, and the co-occurrence pattern was included was included in materials and methods.

c. Multi-way interactions and how they may be shown using network analysis: The authors propose network analysis as a tool to identify higher-order interactions between inversions. However, they do not explain how network analysis achieves this. A simple illustrative example, such as what results would look like under a hypothetical 3-way interaction, would greatly aid reader comprehension.

**A/:** Analysis included now in the paper to detect third and fourth order motifs through the networks. Thank you for the suggestion.

d. Binarized analyses:
i. SD and its downstream effects: When assessing the effects of SD on epistatic interactions, the authors use a binary variable (significant/non-significant SD) as the predictor and count of significant/non-significant interactions (along with mean p-values across combinations) as the independent variable. This choice is not explained – an easy alternative could have been to analyze raw *J* scores using raw SD values as a predictor. If a relationship did exist, residuals extracted from this model could have provided a measure of co-occurrence while controlling for SD. It is possible that I am overlooking a statistical obstacle in proposing this – but such an issue would be worth explaining.
ii. Metrics congruence: The authors use a similar approach to demonstrate congruence between the metrics; however, this congruence could just as easily be assessed using the raw metric scores. Although thematically linked to the rest of this major issue, this point is not a major concern. Here, there is some justification provided; this allows to test if significant hits between the various metrics are robust – but general congruence between these systems would also support the selection of a single system to base the remaining results upon. Additionally, they do compare residuals from these estimates using linear regressions.

**A/:** Excellent suggestion. The binarized step can be omitted, given that the strong relation evinced in the regression analysis is enough and nuanced to both demonstrate the congruence between methods and the justification of moving forward with only one of them in the visualization and network analysis steps.

e. Bootstrapped analyses: For several statistical tests, the authors bootstrap-sample their data to conduct their analysis. While there is nothing inherently wrong with this approach, the manuscript would benefit from a clear rationale for its use. For example, when estimating co-occurrence metrics and their p-values, it seems feasible to perform the calculations directly on the full dataset without resampling. Additionally, while bootstrapping should yield a distribution of p-values, the authors present their significant/non-significant binarization (for testing metric congruence and relationship with SD) as if they are working with a single p-value. This approach is not described, and the process for summarizing or selecting p-values requires clarification.

**A/:** Thank you so much for bringing up such an important element. Clarifications were provide and the binarized analyzed was also removed, given that is redundant with the results directly derived from the regression analysis between metrics.

f. Simulated p-value distributions: The authors conduct simulations to account for potential complications arising from Mendelian inheritance. In addition to null simulations, they simulate scenarios with epistatic interactions to illustrate the expected outcomes for their metrics. However, they select rather extreme selection coefficients without providing justification for this choice. It appears these values were chosen to ensure that deviations from additivity would be detectable by their metrics, thereby highlighting the relevant submatrices being affected. If this was indeed the rationale, it should be explicitly stated to clarify the reasoning behind their approach.

**A/:** We clarified the rationale under the selection of such coefficients. Thank you so much for the suggestion. Now it can be seen:

“These specific survival rates (0.2 for deflation, and 1.0/0.7 for inflation/deflation) were deliberately chosen to be pronounced in order to clearly demonstrate the ability of our statistical frameworks to detect deviations from additivity. While such extreme values may be less common in natural populations, they serve an important methodological purpose in validating our analytical approach by producing clear signals that highlight which submatrices are affected under different scenarios of non-additive interactions. This allows us to establish proof-of-principle for our methods before applying them to real data, where the deviations may be more subtle.”

g. p-value corrections: The authors report their method of p-value correction to account for multiple testing, but in the very next sentence explain that they discard these corrections as they are too stringent. This suggests that the remainder of their analysis is likely vulnerable to type-1 errors.

**A/:** Type I errors are indeed a vulnerability of the analysis. We tried several approaches in this regard, but even the least stringent corrections and FDR control were unable to accommodate the complex structure and dependency within our data. Future efforts must be made in order to design a more appropriate correction method, which should take in consideration the boundaries of the p-values obtained through permutational or bootstrap approaches, the non-independency between tests derived from the same 3x3 contingency table, and most importantly, the non-uniformity of the p-value distribution after the midpoint 0.5, which is essential in the estimation of p0 in FDR methods like the q-value correction. Nevertheless, applied as exploratory analysis to detect associations, these pipelines offer a way of selecting targets for further inspection and statistical testing.

**Minor issues**

1. The authors identify that the genetic family / cross identity is an explanatory variable in determining epistasis and the direction thereof. One possible route to such a result could be poor randomization – for instance, if the offspring of each cross was treated together / maintained in the same location of a greenhouse etc. In this context, it would be valuable to note down the systems of randomization used to ensure that this was not the cause for the effects observed. More generally, it would be useful to for the authors to speculate – why is the genetic family so critical here?

**A/:** The plants from all the crosses were grown simultaneously and fully randomized. In addition to randomization, all plants were rotated in location every two days to eliminate greenhouse position effects. The full experimental design is described in [citation to PLoS Genetics paper] and summarized on lines xxx of this paper. Differences among crosses are inevitable mainly because different polymorphisms segregated in each cross.2. Figure 3 is too pixelated to follow – even digitally. Figure labels are mismatched with the text, see lines 144, 149 for instance. Figure captions repeatedly mention the source of the populations used – but this isn’t necessary. Additionally, overall figure titles for supplemental figures would be helpful to distinguish plots from various simulations.

**A/:** Figure was removed from paper. Same take-home message can be derived from the linear models between measurements.

3. Line 123: “Each inversion is split in two entities, homozygous and heterozygous states” – the phrasing here is unclear. Doesn’t this simply mean that each locus is coded as Std, Inv, or Het?

**A/:** Yes. The wording of that paragraph was clarified.

4. Line 133: “We also categorized the pattern of selection in case of SG*… proposed by Fu et al. (2020).” – The term SG appears to be a typo and should read SD.

**A/:** Yes, it is a typo. Thank you so much. It was fixed.

5. Line 134: “The segregation distortion value (SDV), defined as the negative decimal logarithm of the SD-associated values.” – The phrasing here is incomplete or awkward. Perhaps rephrase as: “The SDV is defined as the negative decimal logarithm of the SD-associated values.” Alternatively, if the sentence is missing a clause, this should be clarified to ensure the definition is complete and comprehensible.

**A/:** The wording of the sentence was fixed, now, it reads: “We used the segregation distortion value (SDV), defined as the negative decimal logarithm of the SD-associated p values, to explore the relation between the individual tests and the pairwise contrasts performed later.”

6. Line 149: “For the former method” – the phrasing here is unclear, as the “former method” was already described as using a different package. Explicitly naming the method would make this section easier to follow and eliminate ambiguity.

**A/:** Fixed.

7. Lines 343-358: “Distinct patterns in frequency of inversion combinations observed”: The authors enumerate frequencies of homozygous-homozygous, homozygous-heterozygous, and heterozygous-heterozygous combinations. Firstly, it would be more informative to express these frequencies as a percentage of the total number of possible valid combinations within the sample. Secondly, the authors ascribe positive or negative tendencies of associations based on very equivocal data—for example, describing 9 negative interactions versus 7 positives as a tendency toward negative interactions seems tenuous. Lastly, the manuscript lacks predictions or post-hoc explanations regarding the biological relevance of these patterns. For instance, is there a specific reason or biological justification for heterozygous-homozygous combinations being more frequent than the others? In fact, is there a reason to believe that these outcomes are not simply stochastic?

**A/:** You are absolutely right. We have no evidence to state a directional bias. Also, under our experimental design and without previous information related to this inversion effect in survival, we cannot say that we expected something concrete in terms of combination categories. Text was fixed accordingly.

8. Line 465: “it could shred* light” should be shed.

**A/:** Yes, thank you.

9. Line 505: “Something that results evident from a quick inspection” could be “something evident.”

**A/:** Fixed following your suggestion.

10. Line 519: “it can be seen a significant effect for the heterogeneity test” could be “can be seen as a”.

**A/:** Fixed following your suggestion.

11. Line 530: “are regions … parts of the genome.” could be “are regions … parts of the genome?”

**A/:** The wording was improved. A question was stated directly to favor the flow of the discussion.

12. Lines 539-544: Can be avoided altogether.

a) “*we calculated pairwise statistics between pairs of inversions on the same chromosome indicated a strong attraction or positive epistasis*” could be “pairwise statistics calculated between …. epistasis”. Additionally, this is rather loose usage of the term epistasis. These clearly co-occur due to physical linkage.
b) Likewise, “*pairwise contrasts between different states of dosage (heterozygous against homozygous) turned out to be strongly repulsive (negative epistasis) … given by the fact that a given plant cannot be heterozygous and homozygous for the same inversion simultaneously*” is also trivial and does not need to be reported.

**A/:** We agree. Modifications made following your suggestions.
