# Supplementary material for: Are you with me? Co-occurrence tests from community ecology can identify positive and negative epistasis between inversions in Mimulus guttatus
Source: PLoS One. 2025 Apr 28;20(4):e0321253. doi: 10.1371/journal.pone.0321253 (PMC12036897; doi:10.1371/journal.pone.0321253)
Supplement: Attachment — Submitted filename: Response to reviewers_Round 2.docx [file pone.0321253.s012.docx]

**Additional Editor Comments:**

The manuscript is now greatly improved and nearing completion. There are a few editorial issues outstanding, the main one concerns the supplemental methods and LD comparison. Rev 2 did not have access to this, which explains some of the comments, but there are still some issues.

**A/:** *All extended methods and supplementary results were carefully inspected and included in submission. We ask to keep them as supplementary elements, given the number of results we already have.*

These are the most compelling. The LD addition is an improvement, “We included a simulation-based contrast to illustrate the performance of these two metrics in relation to the LD framework” but I think this should be described inthe manuscript (see rev 2). The approach is described in the supplemental section, but I suggest moving it to the main MM. (“Comparison between LD and co-occurrence metrics in an allele-based analysis… fixed association strength of 0.5.”).

**A/:** *We would prefer to maintain the simulation exercise for contrasting LD measurements and co-occurrence metrics as supplementary materials. These demonstration is based on hypothetical inversions, analyzed from the allele frequency perspective, to make them comparable with LD metrics. Additionally, the use of co-occurrence metrics in the manuscript is directed to genotypic frequencies.*

And then include a brief summary, like in the RespRev section, to describe the main findings in the manuscript proper. Also, the results of these analyses (points 1-4) in supplemental section, do not include figures or data. Suggest you add that (like 4. “Heatmap plot showing…”). Finally, please define EDF.

**A/:** *The supplementary materials containing these results were improved in organization. Also, EDF (effective degrees of freedom) was defined in the Extended Methods.*

Second main thing is language, it needs improvement. We strongly encourage the senior author to read and copy edit the MS thoroughly in this edition. At PLOS it is not in the reviewers or editors role to improve the language.

**A/:** *Senior and junior scientists went over the language one more time to improve it. Thank you so much for the suggestions.*

**Intermediate**
The annotations of columns, terms and p values still varies from table to table, and in tables and the footings. Please carefully check all tables, tidy this up and standardize.

**A/:** *The annotation of the columns was standardized. Thank you so much for the suggestion.*

Can you recode large and small numbers, e.g. from 3.29E-30 to more classical annotation 3.29 x 10-30
In text, tables and figures.

**A/:** *Scientific notation changed to classical representation.*

Minor points:
Line 17. Maybe insert “unlinked” into the sentence?

**A/:** *Done.*

Line 56. “Co-occurrence in geneticS…”

**A/:** *Corrected.*

Line 68 should this be singular or plural, one method or many?

**A/:** *Changed to “These metrics …”*

Line 78 on not of “of 4.5mb on chromosome 6”

**A/:** *Corrected.*

Line 83 sentence should end with a citation!

**A/:** *Citation included*.

Line 88 pleasre rephrase “Network analysis is an area of active development in biology [20,21].”
Line 203 which metrics ? “between two widely used LD metrics”

**A/:** *Changes were implemented according to your suggestions.*

Line 206. Figure 2. Is the coupling case – was it deliberate to have one individual of the “fourth type”? In the figure as it “stands”, column 5, line 2.

**A/:** *We do not understand the question.*

Legend of table 1, same X2 annotation for both, no explanation of which is which.

**A/:** *The issue was corrected.*

Is table 2 justified? Is another representation of the data more helping. This appears a bit like a supplemental table, or is it a list of interesting things?

**A/:** *Yes, those inversion genotype pairs are one of the main results of the paper. A set of potential combination with coupling and repulsion effects worth further investigation.*

Figure 3
The scale of X is confusing, can you improve it?

**A/:** *The scale was changed by using classical scientific notation.*

Line 377. “While most inversion pairs show no evidence of interaction, there are interesting exceptions.” Is there maybe more important summary statement at start. Are there different types of patterns to the associations??

**A/:** *The text was rearranged to present the summary with the different patterns of association between dosages levels first.*

Line 387
“The most frequent deviations from independence were observed in heterozygous-homozygous combinations (~58%),” Is this an excess over the expectations. Which fraction of the combinations tested are het-homo? Consider same descriptions for other combinations.

**A/:** *We did not test for an excess of combinations in this case. We did not have a reason to do it. It could be worth it with further data, to see if there is a consistent bias. In terms of proportions, given that for any given pair of inversion, you have two dosage levels in consideration (heterozygous and homozygous), you would have two contrasts heterozygous-homozygous, one heterozygous-heterozygous, and one homozygous-homozygous (i. e. Inv_1_1 vs Inv_2_2; Inv_1_2 vs Inv_2_1; Inv_1_1 vs Inv_2_1; Inv_1_2 vs Inv_2_2). So, ½ of the contrast are heterozygous-homozygous. We did not report again the proportion relative to all the contrast performed because we presented the information related to how many relevant tests from the total we were interested in, and now we say, from those potential contrasts, which ones reflect a deviation from independence in terms of dosage combinations.*

Line 399
Fig missing “as depicted in 3S”?

**A/:** *Corrected.*

Line 411
“S trongest signals were more abundant in lines 155, 502, 541, 62, 664, and 909, while in the rest they were scarcer.” Does this rhyme with the JT analyses?

**A/:** *Yes, in the context of the paragraph, that opens the presentation of the networks per cross.*

Check title supplemental data file “suplemental”
Also, from supplemental methods file, please cross reference to the manuscript for particulars (methods, tables, figures).

**A/:** *Everything was reviewed and cross-referenced.*

**Reviewer 2**

**Major issue #1:** Engagement with LD, comparative analysis.

As far as I can see, neither simulation methods/parameters nor the calculations used to make

subsequent measurements are described in the supplement. I am unable to follow what has been

reported.

**A/:** *The supplementary materials and extended methods were better labeled for identification in the current submission.*

**Major issue #2:** Insufficient explanations

The authors add a description in the introduction2, and a toy example as promised through fig.2.

However, the status of the 'Extended Methods' section is unclear. I am not able to locate such a

section in the “supporting information” file. Perhaps this was intended as a supplementary

section but omitted accidentally.

As noted above, the extended methods were not available for review.

**A/:** *The supplementary materials and extended methods were better labeled for identification in the current submission.*
